# Controlling brain dynamics: Landscape and transition path for working memory

**DOI:** 10.1371/journal.pcbi.1011446

**Published:** 2023-09-05

**Authors:** Leijun Ye, Jianfeng Feng, Chunhe Li

**Affiliations:** 1 Institute of Science and Technology for Brain-Inspired Intelligence, Fudan University, Shanghai, China; 2 Shanghai Center for Mathematical Sciences, Fudan University, Shanghai, China; 3 Department of Computer Science, University of Warwick, Coventry, United Kingdom; 4 School of Mathematical Sciences and MOE Frontiers Center for Brain Science, Fudan University, Shanghai, China; Bournemouth University, UNITED KINGDOM

## Abstract

Understanding the underlying dynamical mechanisms of the brain and controlling it is a crucial issue in brain science. The energy landscape and transition path approach provides a possible route to address these challenges. Here, taking working memory as an example, we quantified its landscape based on a large-scale macaque model. The working memory function is governed by the change of landscape and brain-wide state switching in response to the task demands. The kinetic transition path reveals that information flow follows the direction of hierarchical structure. Importantly, we propose a landscape control approach to manipulate brain state transition by modulating external stimulation or inter-areal connectivity, demonstrating the crucial roles of associative areas, especially prefrontal and parietal cortical areas in working memory performance. Our findings provide new insights into the dynamical mechanism of cognitive function, and the landscape control approach helps to develop therapeutic strategies for brain disorders.

## Author summary

A fundamental aspect of brain science is understanding our brain and interacting with it. Here, using a large-scale model of macaque brain, we investigate the stochastic dynamical mechanism of working memory. The alteration in landscape and brain state switching in response to task demands governs the working memory function. The kinetic transition path demonstrates that the hierarchical structure determines the direction of information flow. Significantly, we propose a landscape control approach to manipulate the relative stability and transition of brain states by identifying key nodes and edges in brain network, to improve the working memory performance. Our findings offer new way to comprehend dynamic mechanism of cognitive function and promote the development of therapeutic strategies for brain disorders.

## Introduction

Working memory is a basic ingredient to many other cognitive functions, such as learning and decision-making. Recently, many studies revealed that persistent neural activity during memory delays can be found in a range of specialized brain areas, from sensory to parietal and prefrontal cortex, suggesting the distributed nature of working memory [1, 2]. However, how these distributed brain regions interact with each other and achieve cognitive function cooperatively remains to be elucidated.

A commonly used way of studying cognition function is to trace the neural activity trajectories in a finite timescale, which mostly reflects the local property rather than the global characteristics of the dynamical working memory system. So is there any possible way to obtain the global natures of collective behaviors emerging in complex neural systems? Inspired by thermodynamics and statistical physics, the collective properties of a complex working memory system composed of multiple interacting elements can be explored by the thermodynamics quantities, such as free energy and entropy. The energy landscape provides a quantitative and intuitive way to investigate the global and transition properties of complex systems. In neuroscience, the concept of “energy function” was first proposed by Hopfield to explore the computational properties of neural circuits, providing a clear dynamical picture of how memory storage and retrieval are implemented [3, 4]. Nonequilibrium potential has been theoretically derived for single neuron models and neural populations, which facilitates understanding the mechanism of neural dynamics [5–7]. Energy landscape was also used in whole-brain modeling to examine the structure-function relationship in the brain [8, 9]. In systems biology, the Waddington landscape has been used as a metaphor for cell development and differentiation [10, 11].

Some efforts have been devoted to understanding the dynamical mechanism of cognitive function from landscape perspective [12–14]. However, these attempts are based on the local neural circuits. For the high-dimensional distributed working memory system with multiple interacting brain areas, how to quantify the landscape and visualize it remains challenging. Furthermore, our brain is inherently noisy. For the storage of working memory, the self-sustained persistent activity during a mnemonic delay is desired to be robust to noise. However, the general principles for maintaining robust working memories in noisy biological systems are unclear.

The brain faces a vast repertoire of ever-changing external and internal stimuli. To respond to these stimuli with appropriate behaviors, the brain dynamics should switch between several possible network states dynamically, rather than settle in one single strong state [15, 16]. The ordered transition between brain states and flexible reconfiguration of brain-wide interactions are essential for the execution of brain function [17]. Altered temporal reconfiguration is also related to some disorders of cortical function such as depression [18] and schizophrenia [19]. For the working memory function, memory can be considered as being stored in the corresponding attractor [20–23]. Several lines of work have also provided support to multiple discrete attractors as the underlying mechanism of decision-making and attention [23–25]. Due to the complexity of brain states, the in-depth study of state transitions has been minimally successful.

The advanced understanding of brain dynamics is often related to our ability to control it, i.e., how to perturb the brain system to reach a desired state. In particular, brain-computer interfaces and neuromodulation technology are two representative applications of controlling our brain, which can alter the dynamics of brain function by changing regional activity. Mathematically, network control theory (NCT) has been capitalized to investigate how the brain switches between cognitive states constrained by structural connectivity [26, 27]. While the neural dynamics are nonlinear, NCT is built on the simplified noise-free linear network model. So how to modulate the brain transition dynamics based on the large-scale nonlinear model poses another challenge.

To address these issues, in this work, based on a large-scale model of distributed working memory [28], we quantified the multistable energy landscape of working memory. When no stimulus is applied, the whole neocortex exhibits three different attractors, characterizing the resting state and two stimulus-selective memory states, respectively. We explored the state transition in working memory function by barrier height inferred from landscape topography and kinetic transition path by minimum action path (MAP) approach. Barrier height quantifies the transition feasibility between memory states against both non-selective random fluctuations and distractor stimuli. Interestingly, the increasing barrier height during the delay epoch can well explain the experimental observation demonstrating that the robustness to distractors is stronger when the distractors are given at the later time of the delay epoch [29]. By quantifying the transition path, we found that the state transition between the two memory states not only passes through the spontaneous state but also shows sequential deactivation from sensory areas to association areas, the memory stored in the cortical area with higher hierarchy is more stable, and information flow follows the direction of hierarchical structure. Finally, we propose a landscape control approach to manipulate the brain state stability and identify the optimal combinations of stimulation targets for improving working memory performance. Our approaches can be applied to study the underlying mechanism of other cognition functions and the brain disorders such as schizophrenia, which are often related to significant cognitive impairment.

## Materials and methods

We provide an overview of the workflow for the energy landscape construction, transition path quantification, and landscape control approach based on the large-scale distributed working memory model.

## The anatomically constrained large-scale model of distributed working memory in macaque

**Fig 1:**
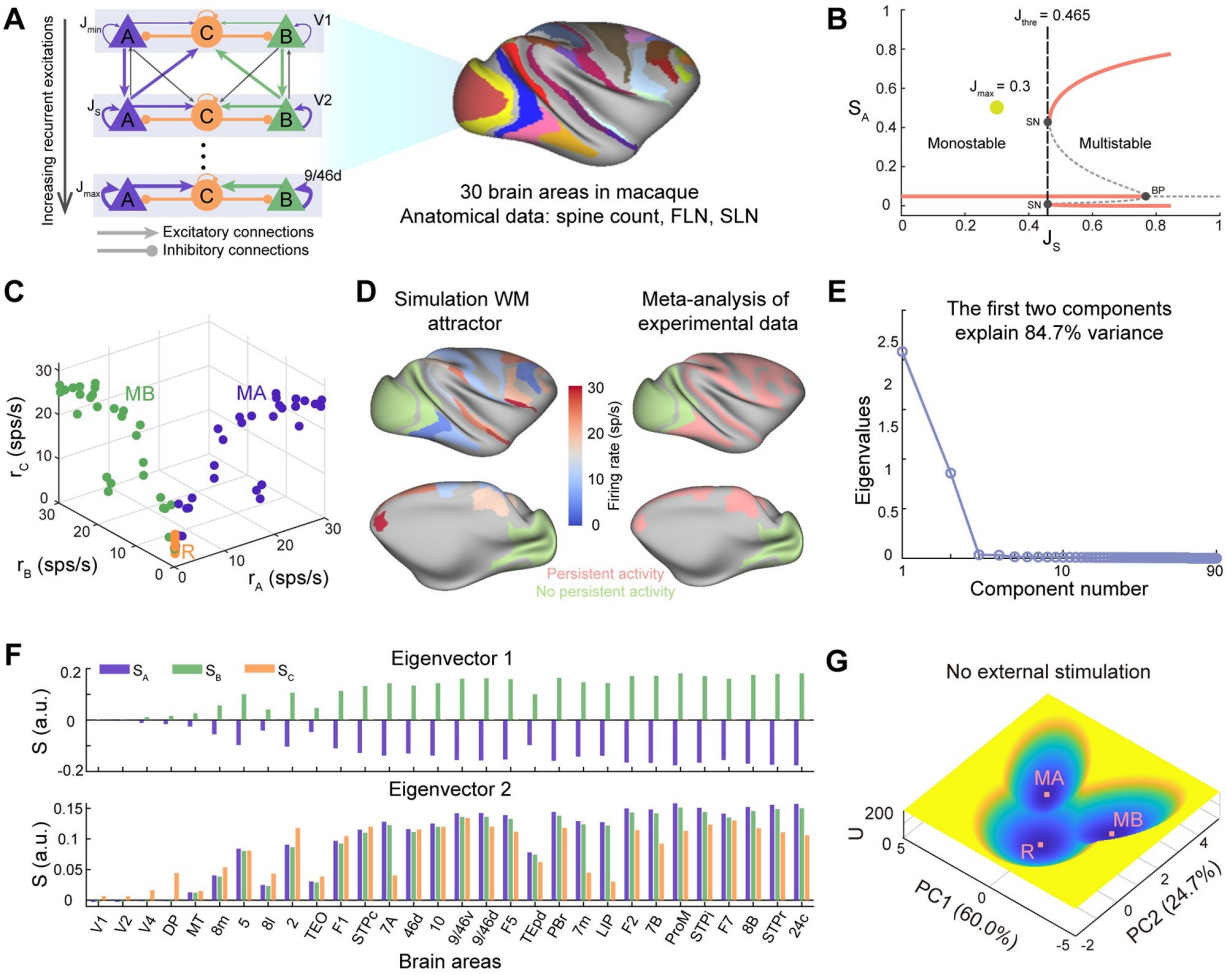
The landscape for large-scale working memory system. (A) The schematic diagram of the computational model of distributed working memory in macaque with 30 brain areas. Each brain area is modeled by two selective excitatory neural populations, labeled as A and B, and one inhibitory population, labeled as C. Each excitatory population has self-excitations and inhibits each other mediated by population C. The inter-areal projections are based on quantitative connectomics data, the fraction of labeled neurons (FLN), and supragranular layered neurons (SLN). For clarity, the cross-couplings between two excitatory populations are not shown. (B) The bifurcation diagram with respect to the recurrent strength (*J*_*S*_) for the isolated area. In this study, unless specified otherwise, we set the maximum recurrent strength (*J*_*max*_) as 0.3, lower than the bifurcation point (*J*_*S*_ = 0.465), so that all the brain areas are monostable when isolated. SN: saddle-node, BP: branch point. (C) The three fixed points of the distributed working memory model, resting state (R), memory state encoded by population A (MA), and memory state encoded by population B (MB). (D) There is a strong overlap between the persistent activity of the memory state predicted by the model and the meta-analysis of experimental data [51]. (E) The eigenvalues of the covariance matrix of the probabilistic distribution of system states for dimension reduction of the landscape. The first two components explain 84.7% of the variance. (F) The eigenvectors corresponding to the first two components. (G) The quantified tristable potential landscape after dimension reduction when no external stimulus is applied. Note that the landscape is symmetric along the PC1 axis. The parameters are chosen as default values.

The mathematical model of distributed working memory we explored here was first proposed and carefully described in [28]. Here we briefly summarize the model. Fig 1A presents the schematic diagram of the large-scale model of the macaque cortex, which engages cortical areas across all four neurocortical lobes, including V1, V2, MT, LIP, V4, 7A, 7m, 8m, 8l, 5, TEO, DP, 2, F1, 7B, TEpd, 10, F5, 46d, PBr, 24c, F2, ProM, STPc, STPi, STPr, F7, 8B, 9/46v, 9/46d. Each cortical area is modeled as the of Wong-Wang model with three fully connected populations [24]. The temporal evolution of the two selective excitatory populations (labeled as A and B) and one inhibitory population (labeled as C) is described by the following equations [28]:

$$\begin{array}{c} \frac{dS_{A}}{dt}=-\frac{S_{A}}{\tau_{N}}+\gamma_{E}(1-S_{A})r_{A}+\zeta_{A}\left(t\right), \end{array}$$ (1)

$$\begin{array}{c} \frac{dS_{B}}{dt}=-\frac{S_{B}}{\tau_{N}}+\gamma_{E}(1-S_{B})r_{B}+\zeta_{B}\left(t\right), \end{array}$$ (2)

$$\begin{array}{c} \frac{dS_{C}}{dt}=-\frac{S_{C}}{\tau_{G}}+\gamma_{I}r_{C}+\zeta_{C}\left(t\right), \end{array}$$ (3)

$$\begin{array}{c} I_{A}=J_{S}S_{A}+J_{C}S_{B}+J_{EI}S_{C}+I_{0A}+I_{net}^{A}, \end{array}$$ (4)

$$\begin{array}{c} I_{B}=J_{C}S_{A}+J_{S}S_{B}+J_{EI}S_{C}+I_{0B}+I_{net}^{B}, \end{array}$$ (5)

$$\begin{array}{c} I_{C}=J_{IE}S_{A}+J_{IE}S_{B}+J_{II}S_{C}+I_{0C}+I_{net}^{C}, \end{array}$$ (6)

$$\begin{array}{c} r_{A,B}\left(I_{A,B}\right)=\frac{aI_{A,B}-b}{1-\text{exp}[-d\left(aI_{A,B}-b\right)]}, \end{array}$$ (7)

$$\begin{array}{c} r_{C}\left(I_{C}\right)=[\frac{1}{g_{I}}(c_{1}I_{C}-c_{0})+r_{0}{]}_{+}. \end{array}$$ (8)

Here, *S*_*A*_ and *S*_*B*_ are interpreted as the fraction of open NMDA-mediated synaptic channels in populations A and B, and *S*_*C*_ is the fraction of open GABAergic-mediated synaptic channel in population C. *I*_*i*_ with i = A, B, C is the synaptic current input to the population ‘i’, which is composed of local inputs from local circuits, background inputs (*I*_0*i*_) and long-range inputs from other areas in the network ($I_{net}^{i}$). *r*_*i*_ is the population-averaged firing rate. The notation [⋅]_+_ in *r*_*C*_ denotes rectification. *ζ*_*i*_ is the Gaussian white noise, introducing stochasticity into the system.

To introduce area-to-area heterogeneity, it is assumed that the local recurrent strength from the early sensory area to the higher association area increases in a gradient way. This is inspired by the anatomical study which identified a gradient of dendritic spine density [30]. In addition, the cortical areas are distributed along an anatomical hierarchy. The anatomical fraction of supragranular layer neuron data is used to build the anatomical hierarchy. The low (high) hierarchy of the source area relative to the target corresponds to the high (low) SLN values of source-to-target projection [31, 32]. Area *i* is assigned a normalized hierarchical value *f*_*i*_ estimated by logistic regression using FLN [32]. More specifically, the local recurrent strength of area *i* is given by

$$\begin{array}{c} J_{S}\left(i\right)=J_{\text{min}}+(J_{\text{max}}-J_{\text{min}})h_{i}, \end{array}$$ (9)

where *h*_*i*_ is the normalized value between zero and one of the dendritic spine count values with age-related corrections observed in anatomical studies. A full list of all area-specific values of spine densities and their sources can be found in Table A in S1 Text. For the missing value of spine count, the anatomical value *f* is used as a proxy due to the high correlation between spine count data and anatomical hierarchy. Therefore, the value of *J*_*S*_ varies from *J*_*min*_ to *J*_*max*_. Furthermore, to ensure that the spontaneous activity is the same for all areas, *J*_*IE*_ also scales with *J*_*S*_:

$$\begin{array}{c} J_{IE}=\frac{1}{2J_{EI}\lambda }(J_{0}-J_{S}-J_{C}), \end{array}$$ (10)

where $\lambda =\frac{\tau_{G}\gamma_{I}c_{1}}{g_{I}-J_{II}\tau_{G}\gamma_{I}c_{1}}$ and *J*_0_ = 0.2112 nA. The minimum value of *J*_*min*_ is 0.205 nA to ensure *J*_*IE*_ is non-negative. In this work, *J*_*min*_ is set to be 0.21. *J*_*max*_ is an important parameter, which determines the type of working memory model. Fig 1B in the main text shows the bifurcation diagram of the isolated area with respect to local coupling *J*_*S*_. When *J*_*max*_ is below the bifurcation point, all areas are monostable in isolation. In this case, it is the inter-areal projections that contribute to the emergence of sustained activity displayed by the model. On the other hand, having *J*_*max*_ above the bifurcation point implies that some higher cognitive areas are intrinsically multistable when isolated, compatible with classical working memory theories.

For inter-areal projections, the quantitative anatomical connectivity data in macaque is available by track-tracing studies [32–34]. The M132 macaque brain atlas including 91 cortical areas is used in this work [34]. The fraction of labeled neurons (FLN) is defined as a proxy of the connection strength between cortical areas. The FLN, as we have discussed before, defines the structural hierarchy of the network. Both FLN and SLN are weighted and directed matrices. These two connectivity data can be downloaded from https://core-nets.org.

Since the inhibitory projections tend to be local, it is assumed that only excitatory populations can generate inter-areal projections. To facilitate the propagation of activity along the hierarchy, it is also assumed that the long-distance outgoing connectivity targets more strongly excitatory populations in a selective way for feedforward pathways and inhibitory populations for feedback pathways (Fig 1A). Then the long-range input to the three populations of area *x* originated from all the other cortical areas *y* is given by

$$\begin{array}{c} I_{A,net}^{x}=G\frac{J_{S}\left(x\right)}{max(J_{S})}\sum_{y}W^{xy}SLN^{xy}S_{A}^{y}, \end{array}$$ (11)

$$\begin{array}{c} I_{B,net}^{x}=G\frac{J_{S}\left(x\right)}{max(J_{S})}\sum_{y}W^{xy}SLN^{xy}S_{B}^{y}, \end{array}$$ (12)

$$\begin{array}{c} I_{C,net}^{x}=\frac{G}{Z}\frac{J_{IE}\left(x\right)}{max(J_{IE})}\sum_{y}W^{xy}(1-SLN^{xy})(S_{A}^{y}+S_{B}^{y}). \end{array}$$ (13)

Here, *G* is the global coupling strength, taken as 0.48 unless specified otherwise. *Z* is a factor balancing long-range excitatory and inhibitory projections, taken as $Z=\frac{2c_{1}\tau_{G}\gamma_{I}J_{EI}}{c_{1}\tau_{G}\gamma_{I}J_{II}-g_{I}}$ to guarantee the net effect of population A and B with the same activity level on other areas is zero. The sum of *y* is for all 30 cortical areas of the network. For population A in a specific area, it is influenced by the A-selective populations of other areas directly and B-selective populations of other areas indirectly via local population C.

Due to the board range of FLN values, FLN is firstly rescaled to a suitable range for the firing rate model by $W_{xy}=k_{1}{\left(FLN^{xy}\right)}^{k_{2}}$ where *k*_1_ = 1.2 and *k*_2_ = 0.3. Then *W* is furthered normalized so that ∑_*y*_ *W*^*xy*^ = 1. Finally, the gradient is also introduced into the long-range projection strengths by multiplying the gradient of *J*_*S*_ for feedforward projections and *J*_*IE*_ for feedback projections. The SLN is taken as the proxy of the feedforward and feedback characteristics of projections. That is, *SLN* = 1 stands for pure feedforward network and *SLN* = 0 represents pure feedback network. So the linear dependence on SLN for projections to excitatory populations and (1-SLN) for projections to inhibitory is also assumed. Inspired by the evidence that frontal networks have primarily strong excitatory loops [35], the SLN-driven modulation of feedback projections from frontal areas to 8l and 8m is modified to be no larger than 0.4. The default values of all parameters and their descriptions are summarized in Table B in S1 Text.

### Quantification of the energy landscape with moment equations

Mathematically, the distributed working memory model can be described by 90-dimensional ordinary differential equations (ODEs):

$$\begin{array}{c} \frac{d\text{x}(t)}{dt}=\text{F}\left(\text{x}\left(t\right)\right), \end{array}$$ (14)

where **x**(*t*) = [*S*_*A*,*V*1_(*t*), *S*_*B*,*V*1_(*t*), *S*_*C*,*V*1_(*t*), …, *S*_*A*,9/46*d*_(*t*), *S*_*B*,9/46*d*_(*t*), *S*_*C*,9/46*d*_(*t*)], representing the time-varying 90-D system variables. **F**(**x**) denotes the driving force of the system. These ODEs describe the temporal evolution of the synaptic current of 90 populations (30 brain areas with 3 populations in each area). For the high dimension and nonlinear dynamical system described above, the numerical solution can be obtained by the Euler’s Method with an integration time step of 0.01*ms*. Furthermore, the time evolution of stochastic dynamics can be expressed in vector form as

$$\begin{array}{c} \frac{d\text{x}(t)}{dt}=\text{F}\left(\text{x}\left(t\right)\right)+\zeta \left(t\right), \end{array}$$ (15)

where *ζ* is a 90-D vector representing independent Gaussian white noise, that is,

$$\begin{array}{c} <\zeta (t)>=0, \end{array}$$ (16)

$$\begin{array}{c} <\zeta \left(t\right)\zeta \left(t^{\prime }\right)>=2d\cdot \text{I}\cdot \delta \left(t-t^{\prime }\right), \end{array}$$ (17)

where **I** is the identity matrix and *δ*(*t*) is Dirac delta function. *d* is the constant diffusion coefficient, characterizing the level of noise. In this work, we choose *d* = 0.1 as the default value.

The corresponding diffusion equation (Fokker–Planck equation) of Eq 15 describing the time evolution of the system probability distribution takes the form as

$$\begin{array}{c} \frac{\partial p(\text{x},t)}{\partial t}=-\sum_{i}\frac{\partial }{\partial x_{i}}[F_{i}\left(\text{x},t\right)p\left(\text{x},t\right)]+d\sum_{i}\sum_{j}\frac{\partial^{2}}{\partial x_{i}\partial x_{j}}p\left(\text{x},t\right), \end{array}$$ (18)

where *p*(**x**, *t*) is the probability density function of system state at time *t*. However, due to the high dimension (90-D) and nonlinearity, the partial differential equation of Eq 18 is intractable. So we assumed that the time evolution of the density function satisfies the Gaussian distribution. When the diffusion coefficient *d* is small, the moment equations describing the time evolution of mean $\bar{\text{x}}\left(t\right)$ and variance matrix **Σ**(*t*) of Gaussian distribution are as follows [36, 37]:

$$\begin{array}{c} \dot{\bar{\text{x}}}\left(t\right)=\text{F}\left[\bar{\text{x}}\left(t\right)\right], \end{array}$$ (19)

$$\begin{array}{c} \dot{\Sigma }\left(t\right)=\Sigma \left(t\right){\text{A}}^{T}\left(t\right)+\text{A}\left(t\right)\Sigma \left(t\right)+2d\cdot \text{I}. \end{array}$$ (20)

Here, **A**(*t*) is the Jacobian matrix of **F**(*x*), i.e., $A_{ij}\left(t\right)=\frac{\partial F_{i}(\bar{\text{x}}\left(t\right)}{\partial x_{j}}$, and **A**^*T*^(*t*) is the transpose of **A**(*t*).

In this work, we focus on the behaviors of the system at the steady state. The probability density distribution of the system at the steady state can be expressed as:

$$\begin{array}{c} p_{ss}\left(\text{x}\right)=\frac{1}{{\left(2\pi \right)}^{\frac{N}{2}}{\left|\Sigma \right|}^{1/2}}\text{exp}\{-\frac{1}{2}{\left(\text{x}-\mu \right)}^{T}\Sigma^{-1}\left(\text{x}-\mu \right)\}, \end{array}$$ (21)

where *μ* and **Σ** are the solutions of Eqs 19 and 20 when *t* → +∞, respectively. Dependent on the initial conditions of the system variable (the conductance of receptors in this work), Eqs 19 and 20 may have different solutions at the steady state, which means that the system may be multistable. In this condition, the final probability density function is determined by the weighted sum of multiple Gaussian distributions, that is,

$$\begin{array}{c} p_{ss}\left(\text{x}\right)=\sum_{j=1}^{M}w_{j}p_{ss}^{j}\left(\text{x}\right). \end{array}$$ (22)

Here, *M* is the number of stable states of the system, $p_{ss}^{j}\left(\cdot \right)$ represents the density function of *j*th stable state taken the form of Eq 21 and *w*_*j*_ denotes the corresponding weight. The weight is estimated by the statistic of frequency of each stable state under abundant initial conditions (10000 initial conditions in this work). The system variables are randomly initialized by sampling from the uniform distribution in the range from 0 to 1.

Finally, the potential landscape at the steady state is defined as *U*(**x**) = −ln *P*_*ss*_(**x**) [10, 38, 39], where *P*_*ss*_ represents the normalized probability distribution at the steady state, and *U* is the dimensionless potential. However, for the high-dimensional system, the high-dimensional potential landscape is hard to visualize and interpret. We used previously developed dimension reduction approach to display the landscape in reduced dimensions [39]. The high-dimensional potential landscape is projected to the 2D subspace spanning by the first two components *PC*1 and *PC*2 (See Section A in S1 Text for details).

### Identification of minimum action paths (MAPs)

For a multistable system, it is of great importance to identify the transition paths between different attractors, which provide information on switching order for different components in the state transition process. Firstly, we define the path between *i*th attractor **x**^*i*^ at time 0 and *j*th attractor **x**^*j*^ at time *T* as ${\text{x}}^{ij}\left(t\right)=(x_{1}^{ij}\left(t\right),x_{2}^{ij}\left(t\right),\cdots ,x_{n}^{ij}\left(t\right){)}^{T}$, *t* ∈ [0, *T*]. Then the path **x**^*ij*^(*t*) satisfies the following boundary conditions:

$$\begin{array}{c} \left\{ \begin{array}{c} (x_{1}^{ij}\left(0\right),x_{2}^{ij}\left(0\right),\cdots ,x_{N}^{ij}\left(0\right){)}^{T}={\text{x}}^{i},\\ (x_{1}^{ij}\left(T\right),x_{2}^{ij}\left(T\right),\cdots ,x_{N}^{ij}\left(T\right){)}^{T}={\text{x}}^{j}. \end{array}\right. \end{array}$$ (23)

Let *L*^*ij*^ be the distance between the driving force **F** and the velocity along the transition path: $L^{ij}(t,{\text{x}}^{ij}\left(t\right),\frac{d{\text{x}}^{ij}\left(t\right)}{dt})=\|(\frac{dx_{1}^{ij}\left(t\right)}{dt},\frac{dx_{2}^{ij}\left(t\right)}{dt},\cdots ,\frac{dx_{N}^{ij}\left(t\right)}{dt}{)}^{T}-\text{F}({\text{x}}^{ij}\left(t\right)){\|}_{2}$. The transition action *S*(**x**^*ij*^) is defined as the integral of the Lagrangian *L*^*ij*^ between time 0 and *T*. Based on Wentzell-Freidlin theory [40], the estimation of the probability distribution of the solution **x**(*t*) over time interval [0, *T*] at a give *δ* is

$$\begin{array}{c} P\{\rho (\text{x}\left(t\right),{\text{x}}^{ij}\left(t\right))<\delta \}\approx \text{exp}\;(-\frac{S_{ij}({\text{x}}^{ij})}{\varepsilon }). \end{array}$$ (24)

So the most probable transition path starting from **x**^*i*^ for *t* = 0 and ending at **x**^*j*^ for *t* = *T* can be obtained by solving the following optimal problem [41–43]:

$$\begin{array}{c} \underset{{\text{x}}^{ij}}{\text{min}}\;S({\text{x}}^{ij})=\frac{1}{2}\;\underset{{\text{x}}^{ij}}{\text{min}}\;\int_{0}^{T}L^{ij}(t,{\text{x}}^{ij}\left(t\right),\frac{d{\text{x}}^{ij}\left(t\right)}{dt})dt. \end{array}$$ (25)

This optimal path is called the minimum action path (MAP), which is also identified as the biological path between attractors. In this work, we calculated the MAPs numerically, using the minimum action methods [42, 44]. The time interval *T* for state transition is an important parameter in the calculation of minimum action. Larger *T* can result in more accurate but unstable results. To find the optimal *T*, we performed the grid search when *T* changes from 0.5 to 500 and finally set 10 as the default value (See Fig D in S1 Text).

### Landscape control from optimization of transition actions

The purpose of landscape control (LC) is to alter the switching behaviors between stable states to improve the working memory function by manipulating tunable parameters Θ. This can be formalized as an optimization problem over the parameters Θ subject to the given constraints [45, 46], i.e.,

$$\begin{array}{c} \underset{\begin{array}{c} \Theta \\ {\left\{g_{k}(\Theta )=\eta_{k}\right\}}_{k}\\ {\left\{h_{k}(\Theta )\leq l_{k}\right\}}_{k} \end{array}}{\text{max}}\;\pi_{m}\left(\Theta ;\varepsilon \right). \end{array}$$ (26)

Here, *π*_*m*_(Θ; *ε*) denotes the occupancy of the memory state with given noise intensity *ε*. *g*_*k*_(Θ) = *η*_*k*_ and *h*_*k*_(Θ) ≤ *l*_*k*_ are introduced to constrain the parameters modified within specific ranges, which are determined by biophysical and experimental constraints. For example, we suppose that there is no bifurcation during the optimization process, which indicates that the complete elimination of undesired states is infeasible. For a system with *N* stable states, the occupancy of stable states can be estimated from the *N* × *N* transition rate matrix **P** with each element described by

$$\begin{array}{c} P_{i,j}^{\varepsilon }=\{\begin{array}{cc} P_{i,j}^{\varepsilon } & i\neq j\\ 1-\sum_{j}P_{i,j}^{\varepsilon } & i=j \end{array}. \end{array}$$ (27)

Here, the transition rate between two different stable states *i* and *j* can be approximated as $P_{i,j}^{\epsilon }\propto exp\left(-\frac{1}{\epsilon }S_{i,j}\right)$ where *ϵ* represents noise strength and *S*_*i*,*j*_ is the minimum of the Freidlin-Wentzell action (Eq 25). It is assumed that the transition process between stable states is Markov, then the limiting occupancy of all stable states *π* can be described by the equilibrium solution of the Markov process, i.e., to solve to equation *π* = *π* **P**. *π*_*m*_ is one element of *π*. The Matlab function “fmincon” was used to perform the optimizations. Due to the optimizations based on the 90-D distributed working memory model are time-consuming and computationally expensive, we employed the simplified version of the original model with 30 excitatory nodes (See Section B in S1 Text for details).

## Results

### Energy landscape construction from large-scale working memory model

The large-scale computational model of distributed working memory (DWM) function we employed here is firstly developed by [28] (schematized in Fig 1A). Different from previous modeling efforts which are restricted to the behaviors of local circuits [47, 48], the DWM model is characterized by the anatomically constrained multi-regional interactions supporting the emergence of distributed persistent activity in working memory function. Each cortical area is modeled as two selective excitatory neural populations, labeled as A and B, and one inhibitory neural population, labeled as C [24]. This subnetwork is fully connected and the winner-take-all competition between populations A and B mediated by GABAergic inhibitory population C results in the stimulus-selective self-sustained persistent activity [24]. The local circuit is placed in 30 areas across all four neocortical lobes and all the cortical areas are interconnected according to the neuroanatomical connectivity matrix FLN. A macroscopic gradient of synaptic excitations is introduced into the model (Table A in S1 Text) [49], that is, both the local recurrent and long-range excitations are area-specific and increase along the cortical hierarchy. The default parameters of DWM model are summarized in Table B in S1 Text.

Firstly, we explore the dynamics of each local circuit, which is also the classical working memory model. From the bifurcation diagram with respect to recurrent excitations (*J*_*S*_)(Fig 1B), there exists a threshold level (*J*_*S*_ = 0.465) separating the monostable dynamics from the multistable dynamics. The monostability means that the system has one exclusively resting state with all the population activity at a low level. Multistability refers to three coexisting stable states, one resting state, and two stimulus-selective persistent activity states with one of the selective populations at high firing activity. In this work, unless specified otherwise, we set *J*_*max*_ = 0.3 lower than the threshold level, that is, all the cortical areas are monostable when isolated. In this situation, the emergence of persistent activity pattern is due to the interactions between cortical areas.

Then we consider the dynamics of distributed working memory function engaging multiple interacting brain regions. The brain is inherently noisy, originating from intrinsic random fluctuations and external distraction [50]. Previous work analyzed individual dynamical trajectories in the finite time scale as well as the phase transition using the bifurcation approach [28]. However, the stochastic transition properties and global stability of working memory systems remain to be quantified. Here, we focus on the probabilistic evolution of the working memory system to explore corresponding stochastic dynamics. Since neural network systems are open systems constantly interacting with the external environment, we resort to the non-equilibrium statistics physics approach. With our landscape and transition path framework (See Methods for details) [10, 38, 39], we can quantify the potential landscape of the attractor dynamics of working memory function and further explore the probabilistic switching process across barriers on the potential landscape to describe the transition dynamics.

Starting from the DWM model, the Langevin equation describing the stochastic dynamics of the working memory system takes the forms of $\frac{d\text{x}(t)}{dt}=\text{F}\left(\text{x}\left(t\right)\right)+\zeta \left(t\right)$ with **ζ** assumed to be Gaussian white noise. The Langevin equation is corresponding to the Fokker-Planck diffusion equation describing the temporal evolution of system probability distribution. It is hard to solve the diffusion equation directly, due to the high dimensionality and nonlinear interactions of the DWM model. By assuming the probabilistic distribution of the system state is Gaussian and determining the mean and variance of Gaussian distribution from moment equations, the temporal evolution of the probabilistic distribution of the system state can be solved numerically (See Methods and Fig A in S1 Text for details). Then, the potential landscape is defined as *U*(**x**) = −ln *P*_*ss*_(**x**) [38] with *P*_*ss*_ representing the probabilistic distribution at steady state. Furthermore, for the visualization of the high-dimensional landscape, we conducted dimension reduction to project the landscape onto the subspace spanning by the first two components [39].

By initializing the DWM model with 10000 different initial conditions, the DWM system shows three stable states (Fig 1C and B in S1 Text) when no stimulus is applied. Here, three different colors indicate three individual stable states. For each stable state, each dot represents one brain area, and its position in three-dimensional space is determined by the stable firing rate of corresponding three populations (*r*_*A*_, *r*_*B*_, and *r*_*C*_). The orange state is identified as the resting state (R) since all three populations in each area are at low spontaneous activity. The purple and green states are symmetrical and identified as the memory state encoded by population A (MA) and the memory state encoded by population B (MB), respectively. For MA, population A and population C of some specific areas show high firing rate while population B keeps in low activity. MB is on the contrary. Of note, the areas displaying activation in MA or MB involve frontal, temporal, and parietal lobes, except early visual areas (V1, V2, V4, DP, MT). This is consistent with the qualitative meta-analysis results concluding that persistent activity during the delay period in working memory tasks is more frequently observed in association areas while reports on early sensory are rare (Fig 1D) [51].

To explore the global stability of multiple attractors, we mapped out the energy landscape of the working memory system and visualized the landscape in two-dimensional space from dimension reduction (Fig 1G) [39]. For the dimension reduction of the landscape, the first two principal components (*PC*1 and *PC*2) have a total contribution rate of 84.7% (Fig 1E), suggesting that projection on PC1 and PC2 preserves well the stability information of the original system. Fig 1F presents the eigenvectors corresponding to the first two eigenvalues, respectively. For eigenvector 1, all *S*_*A*_ in 30 brain areas are less than zero, *S*_*B*_ are larger than zeros and *S*_*C*_ are nearly zero. So *PC*1 reflects which selective population (A or B) shows high firing rate. Eigenvector 2 mainly indicates whether the inhibitory populations C are at the high firing level or not. The potential landscape of distributed working memory system after dimension reduction exhibits three attractors in the absence of external input (Fig 1G). Each attractor is surrounded by its own basins of attraction (the dark blue region) and basins are separated by basin boundaries. By combining the characteristics of the two eigenvectors and high-dimensional stable states, it is easy to infer which high-dimensional stable state the three low-dimensional attractors after dimension reduction correspond to, respectively. The basin with both *PC*1 and *PC*2 approximating zero is identified as the R state. The remaining two symmetrical attractors are memory states. Basin with *PC*1 < 0 is for MA and *PC*1 > 0 for MB. Compared to the resting state, populations C in 30 brain areas show high firing rate in both two memory states, suggesting the winner-take-all competition between two excitatory populations is mediated by the inhibitory population. Of note, even without external input, the presence of noise can drive the probabilistic jumping across barriers between attractors, leading to the state transition between different attractors. Thus, the landscape provides a framework to explore stability and the stochastic transition dynamics of the working memory function.

### Landscape results explain the working memory function with multistable attractors

To meet the demands of external cognitive tasks, the working memory system needs to reconfigure itself, and the system state changes over time [17]. For example, a new stimulus can destabilize the present attractor and may drive the system to another attractor corresponding to the incoming stimulus. Furthermore, the failure to engage the optimal system state in a timely manner is associated with poorer task performance [17, 23]. In the attractor landscape framework, we can explicitly quantify the underlying potential landscape performing the distributed working memory function in a global way.

**Fig 2:**
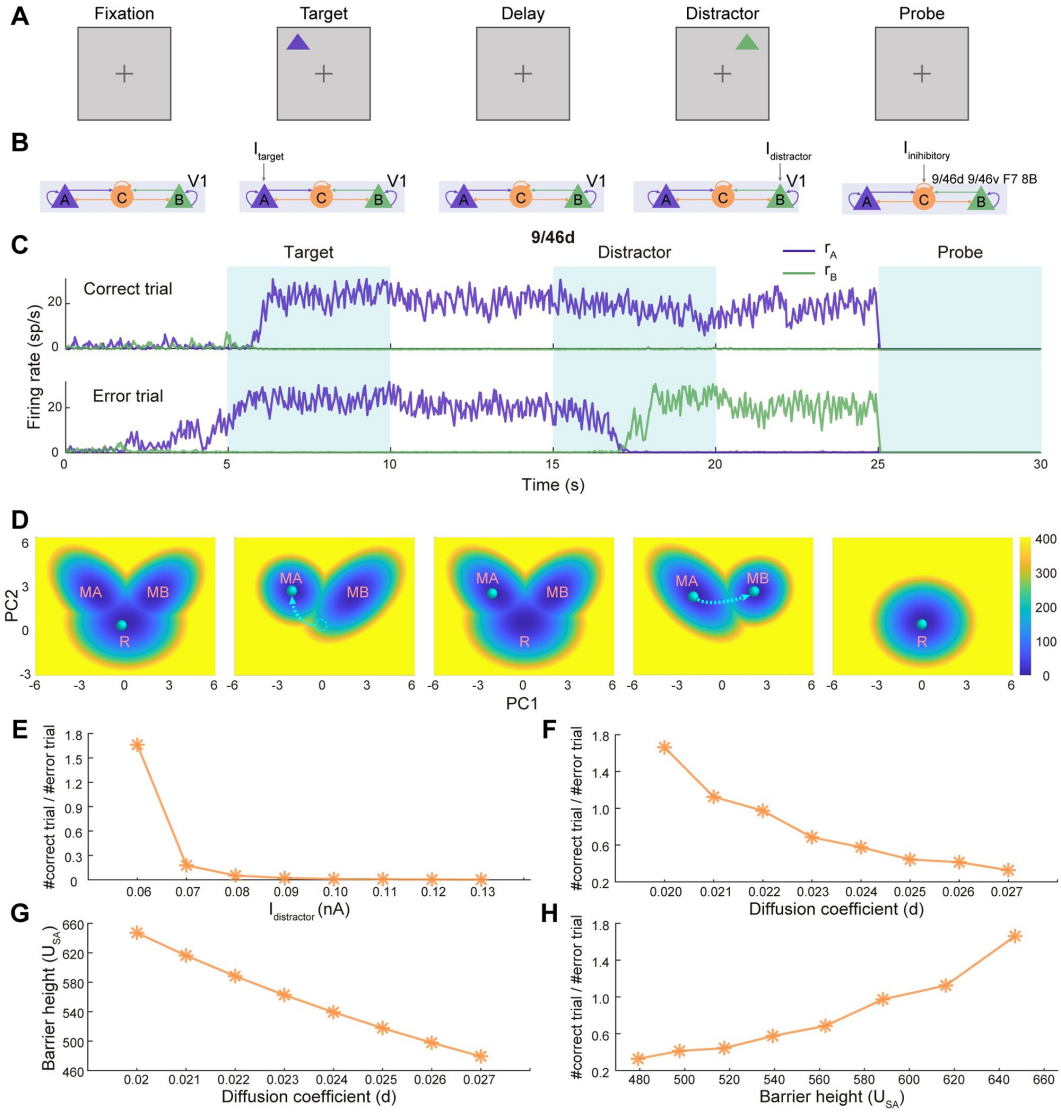
Landscapes explain the working memory function with multistable attractors. (A-B) The schematic illustration of the visual working memory task. During the fixation phase, no external stimulus is applied. During the target phase, a cue is given, which is modeled as the population A of V1 receiving external current. Then the stimulus is removed and the target is expected to be maintained in working memory against random fluctuations and distractors encoded by population B at the delay epoch. Once the task has been done, the sustained activity should be shut down by delivering excitatory input to the inhibitory populations in four areas (9/46d, 9/46v, F7, 8B), leading to the clearance of working memory. (C) The stochastic neural activity traces of selected areas for correct and error trials (See Fig C in S1 Text for behaviors of all areas). For correct trial, the target stimulus is maintained at delay epoch against distractor. On the contrary, the distractor induces the transition from the stimulus-selective high activity state to the distractor-selective high state for error trial. (D) The corresponding attractor landscapes during different phases of the working memory task. The green ball represents the system state. Before the stimulus onset, the system stays at the resting state (R) with all the populations at low activity. The stimulus changes the landscape topography from tristability to bistability. And the system state transits from R to the dominated target-related memory state (MA). Even after the withdrawal of stimulus and the landscape topography returns back to tristability, the system state keeps staying at MA. However, the presentation of distractor stimulus changes the landscape again and may or may not induce the transition to distractor-related attractor (MB), corresponding error and correct trials, respectively. (E-F) The dependence of the proportion between correct and error trials on the intensity of distractor input and the diffusion coefficient. (G) The barrier height between saddle point and MA (*U*_*SA*_) versus the diffusion coefficient. (H) The proportion between correct and error trials versus *U*_*SA*_.

We modeled different phases of the working memory task (Fig 2A) with the DWM model (Fig 2B). During the target phase, a visual cue is given, which is modeled as the population A of V1 receiving external current (*I*_*stimulus*_) [28]. Then the stimulus is removed and the target is expected to be maintained in working memory against random fluctuations and distractors encoded by population B (*I*_*distractor*_) at the delay epoch. Once the action has been performed, the sustained activity should be shut down by delivering input (*I*_*inhibitory*_) to the inhibitory populations in the top four areas of the hierarchy (9/46d, 9/46v, F7, 8B) [28], leading to the clearance of working memory. In Fig 2C and 2D, dependent on the phase of the task, the dynamics of distributed working memory system are described by the time-evolving firing rate of neural populations in representative brain areas 9/46d (See Fig C in S1 Text for trajectories in more areas) and the corresponding potential landscape (See Fig B in S1 Text for coexisting high-dimensional stable states and corresponding weight under different task phase). The green ball represents the state of the working memory system (Fig 2D). We argue that the landscape provides a quantitative and global explanation for the mechanism of working memory function at different stages through state transition dynamics (Fig 2D). In the fixation period, since no stimulus is applied, the system stays at the resting state with all the populations at the low activity (Fig 2D, the first column). In the target period, the target visual stimulus changes the landscape topography from tristability to bistability and the symmetry of the landscape is broken. The MA state becomes dominant by deeper basins of attraction while the basin of the R state disappears (Fig 2D, the second column). When the stimulus is strong enough, the system transits from R to the MA state. Even after the withdrawal of stimulus in the delay epoch and the landscape returns back to tristability, the system can be maintained at the MA state since the MA is a stable attractor (the third column of Fig 2A, 2B and 2D). So, the landscape attractor guarantees the stability of the working memory state. However, the distractor stimulus can change the landscape topography again and the MB state becomes dominant (Fig 2D, the fourth column). Then there will be two outcomes. The system can be either distracted, switching to MB, or not distracted, keeping staying at MA, corresponding to the error and correct trials, respectively (Fig 2C). At the end of the task, the inhibitory input changes the landscape topography to monostability and the system state returns back to the resting state (the last column of Fig 2A, 2B, 2C and 2D).

**Fig 3:**
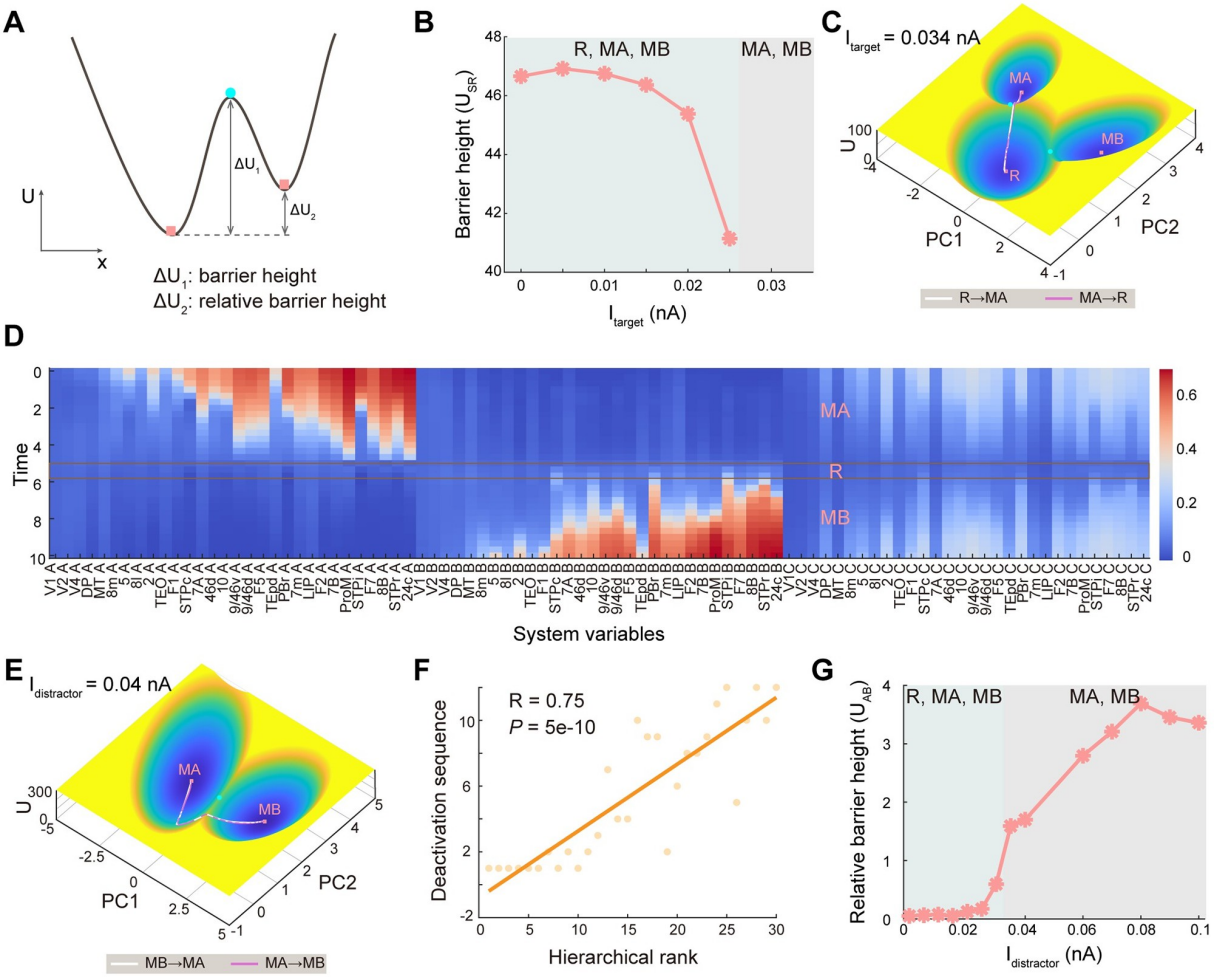
Barrier height and transition path characterize the switching process in working memory. (A) The illustration for the definition of barrier height (potential difference between the potential at local minimum and saddle point) and relative barrier height (the difference between the two barrier heights) to quantify the stability of attractors. If the system tries to escape from the current attractor, it needs to cross the corresponding barrier. (B) To quantify the difficulty of state transition for the formation of memory, we define the barrier height *U*_*SR*_ as the potential difference between the *U*_*saddle*_ (the potential at the saddle point between R and MA state) and the *U*_*min*_ (the potential at the minimum of R state). The *U*_*SR*_ decreases for higher strength of target stimulus, suggesting the easier formation of memory. (C) The projected transition path with dimension reduction on the landscape between R state and MA state for *I*_*target*_ = 0.034*nA*. The transition from R to MA represents the formation of memory and the reverse path represents the clearance of memory. (D) The high-dimensional transition path from MA to MB before dimension reduction for a given time *T* = 10. The X-axis represents the 90 system variables and the Y-axis represents the time points along the transition path. (E) The transition paths between MA and MB for *I*_*distractor*_ = 0.04*nA*, which pass through the intermediate state R. The transition from MA to MB signifies the change of memory. (F) The deactivation sequence of population A in 30 brain areas shows a high correlation with the anatomical hierarchy as defined by layer-dependent connections [32]. This suggests that the information of distractors flows along the hierarchy, from early sensory areas to association areas. (G) The robustness against distractors during the delay epoch. The increase of relative barrier height between MA and MB as a function of the strength of distractors suggests that MA is becoming more unstable while MB is becoming more stable, thus decreasing the robustness of the system to distractors. R: resting state, MA: memory state encoded by population A, MB: memory state encoded by population B.

It is also interesting to explore the proportion between correct and error trials in our model. Specifically, two factors are considered: the intensity of distractor input (*I*_*distractor*_) and the diffusion coefficient (*d*), which reflects the level of noise in the system. We found that as these factors increased, there was a decrease in the proportion of correct trials (Fig 2E and 2F). This finding aligns with our intuitive understanding, as higher distractions and increased noise tend to hamper task performance. From the landscape perspective, we defined barrier height *U*_*SA*_ (potential difference between saddle points and the local minimum of MA) to measure the stability of stimulus input and the difficulty of state transitions when the system is distracted (See Fig 3A for the illustration of barrier height). The increasing noise corresponds to lower *U*_*SA*_ (Fig 2G), thus inducing more transitions from MA to MB in the distractor epoch. Conversely, when the barrier height was higher (indicating a more difficult state transition), we observed a higher proportion of correct trials (Fig 2H). These findings suggest that the barrier height quantified from the landscape provides valuable insights into the relationship between the system’s stability, the difficulty of state transitions, and subsequent behavioral performance. It serves as a useful metric for understanding and predicting task outcomes in scenarios involving distractions and noise-induced influences.

### Transition paths characterize the dynamical switching process in working memory

For the multistable systems including the distributed working memory network, the system may hop between different stable states driven by external input and noise, which corresponds to the alteration of cognitive states [17, 52, 53]. For the working memory task, the switch from R to MA signifies the formation of target-related memory and the switch from MA to MB represents the alteration of memory to the distractor-related state. The state transition is associated with the stability of the corresponding state. An advantage of the landscape approach is that the stability of each attractor can be quantified through the height of the separating barriers. We provide an illustration diagram for the definition of barrier height to quantify the stability of attractors (Fig 3A). Specifically, the barrier height is defined as the difference between *U*_*saddle*_ (the potential at the unstable saddle point separating the basins of attraction for two neighboring attractors) and *U*_*min*_ (the potential at a local minimum of the basin of attraction), which reflects the depth of the basin or attractor. Further, we can define relative barrier height as the potential difference between two barrier heights to measure the relative stability between attractors.

During the target epoch in the working memory task, we found that with the increase in the strength of the target stimulus, the landscape shows a phase transition from tristability (coexisting R, MA, and MB states) to bistability (coexisting MA and MB state). Barrier height *U*_*SR*_ is defined as *U*_*SR*_ = *U*_*saddle*_ − *U*_*R*_ (*U*_*saddle*_ is the potential at the saddle point between R and MA states and *U*_*R*_ represents the potential minimum at R state), quantifying the stability of R state. The decaying trend of *U*_*SR*_ indicates that the R state becomes more and more unstable with the increase of target strength until disappears and becomes the unstable saddle point between MA and MB (Fig 3B).

To quantify the dynamical process for the working memory function, a natural question arises as to what is the biological path during state transition, which will include the information for the activation order of different brain regions during the transition process. Mathematically, the most probable transition paths between stable states can be identified by minimizing the action functional associated with all possible paths connecting initial and final states over a specified time interval of transition [41–43]. For the equilibrium (gradient) system, the minimum action paths (MAPs) have to go through saddle points. However, for the non-equilibrium working memory system we studied here, the MAPs will deviate from the saddle point (See the MAPs between R and MA state in Fig 3C) due to another force, curl flux [38].

Working memory is desired to be robust to the distractor. The distractor occasionally switches the population dynamics to the distractor-related attractor, followed by incorrect actions. We next analyzed the dynamic properties associated with switching from the stimulus-related state (MA) to the distractor-related state (MB) under the perturbation of distractors. Both the 90-D transition path (Fig 3D) and the 2-D path after dimension reduction (Fig 3E) reveal that the transition between MA and MB requires passing through R. That is, the former memory needs to be erased first (backing to the resting state) and then a new memory state forms. MA does not suddenly shift to MB without first accessing a state associated with an intermediate cognitive demand. Interestingly, there is a strong positive correlation (r = 0.75) between the cortical hierarchy [32] and the sequence of deactivation (from high to low firing rate, identified from MAPs (Fig 3D)) of brain areas during state transition (Fig 3F). This suggests that memory is more robust in association areas compared to early sensory areas, and information flow seems to follow the direction of hierarchical structure. This reminds us of the hierarchy of intrinsic timescales across cortical areas [54, 55]. The primary sensory areas should have a faster timescale to encode and process changing external stimuli rapidly while the timescale of prefrontal and parietal cortical areas is larger to accumulate and integrate information for higher cognitive function. The more robust memory in the association area may result from the longer timescale.

We also found that with the increase of the amplitudes of distractor stimulus, the relative barrier height between MA and MB (*U*_*AB*_ = *U*_*MA*,*min*_ − *U*_*MB*,*min*_) increases, i.e., the MA attractor becomes less stable and the MB attractor becomes more stable (Fig 3G). Thus, the state transition from MA to MB is easier, leading to weaker robustness against distractors and poorer behavioral performance.

### The mechanism of temporal gating of distractors in the delay epoch

Since there is a temporal separation between sensation and action in the working memory task, to protect the memory against interferences, any additional inputs arriving during the delay epoch need to be effectively gated. As discussed before, the greater strength of distractors is harder to gate. However, recent work reveals that the gating of distractors depends on not only the strength of distractors but also the timing that distractors arrive at [29]. They found that compared to distractors presented during sample or early delay, distractors presented late in the delay epoch have less impact on behaviors. That is, distracting stimuli arriving in the delayed epoch become less capable of influencing previous memory as the time to act approaches. As shown below, our landscape approach provides a quantitative explanation for the mechanism of the temporal gating of distractors.

**Fig 4:**
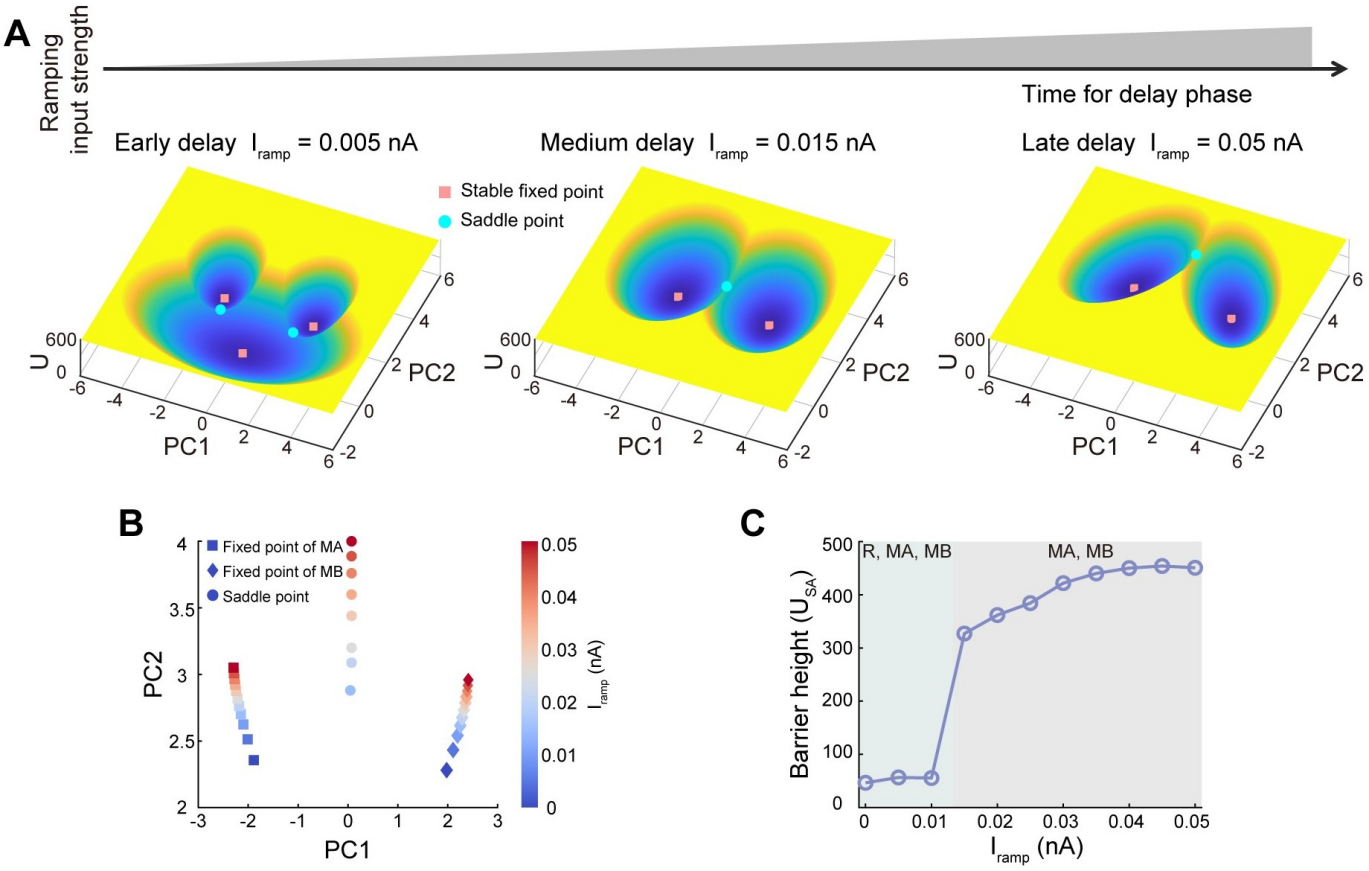
Barrier height explains the mechanism of temporal gating of distractors in delay epoch. (A) The time in the delay epoch is modeled as the non-selective ramping external input to all populations in the system [23, 29, 56]. Three typical landscapes are presented to illustrate the effect of external ramping input on the attraction basins, corresponding to the distractor delivered at the early, medium, and late time of the delay epoch. (B) The two fixed points for memory states moved further away from each other for stronger ramping input *I*_*ramp*_. (C) The barrier height (*U*_*SA*_), defined as the potential difference between the saddle point and the fixed point of MA, increases with increasing ramping input. For weaker ramping input, the distractors can more easily push the system state beyond the saddle point and switch from the target-related attractor to the distractor-related attractor.

Experimental studies revealed that in the mice delayed response task, a fraction of neurons in the premotor cortex exhibits non-selective slow ramping activity [23, 56]. Further observations suggest that this particular ramping reflects the animal’s expectation about timing in the task [29, 56]. So in this work, task-relevant timing is modeled by a continuous, nonspecific ramping input (*I*_*ramp*_) to all neural populations in the DWM. We quantified three typical attractor landscapes for distractors delivered at the early, medium, and late time of the delay epoch (Fig 4A). For the increase of ramping input (i.e., the later time of the delay period), the landscape first shows a phase transition from tristability to bistability and then a progressive separation of attraction basins that encode alternative memories. The results of fixed points and saddle points suggest that the two fixed points move further apart from each other and the distance between the fixed point and saddle point increases as the ramping input increases during the delay epoch (Fig 4B). This agrees well with previous results based on the Recurrent Neural Network (RNN) [23]. Quantitatively, we define barrier height *U*_*SA*_ = *U*_*S*_ − *U*_*MA*_ to quantify the stability of MA, where *U*_*S*_ represents the potential at the saddle point formed by MA and its neighboring attractor (R state for tristability and MB state for bistability) and *U*_*MA*_ denotes the potential at the minimum of MA attractor. The barrier height is higher for stronger ramping input (Fig 4C), indicating that ramping input increases the difficulty of jumping from MA (target-related attractor) to MB (distractor-related attractor) by elevating the corresponding barriers. As a consequence, distractors are more likely to induce a switch between attractors under weaker ramping input compared with stronger input. Therefore, increasing the barrier height of landscape topography results in the filtering of distractors, which provides a quantitative explanation for experimental observations [29].

### The influence of activation and inhibition strengths on the stability of working memory

**Fig 5:**
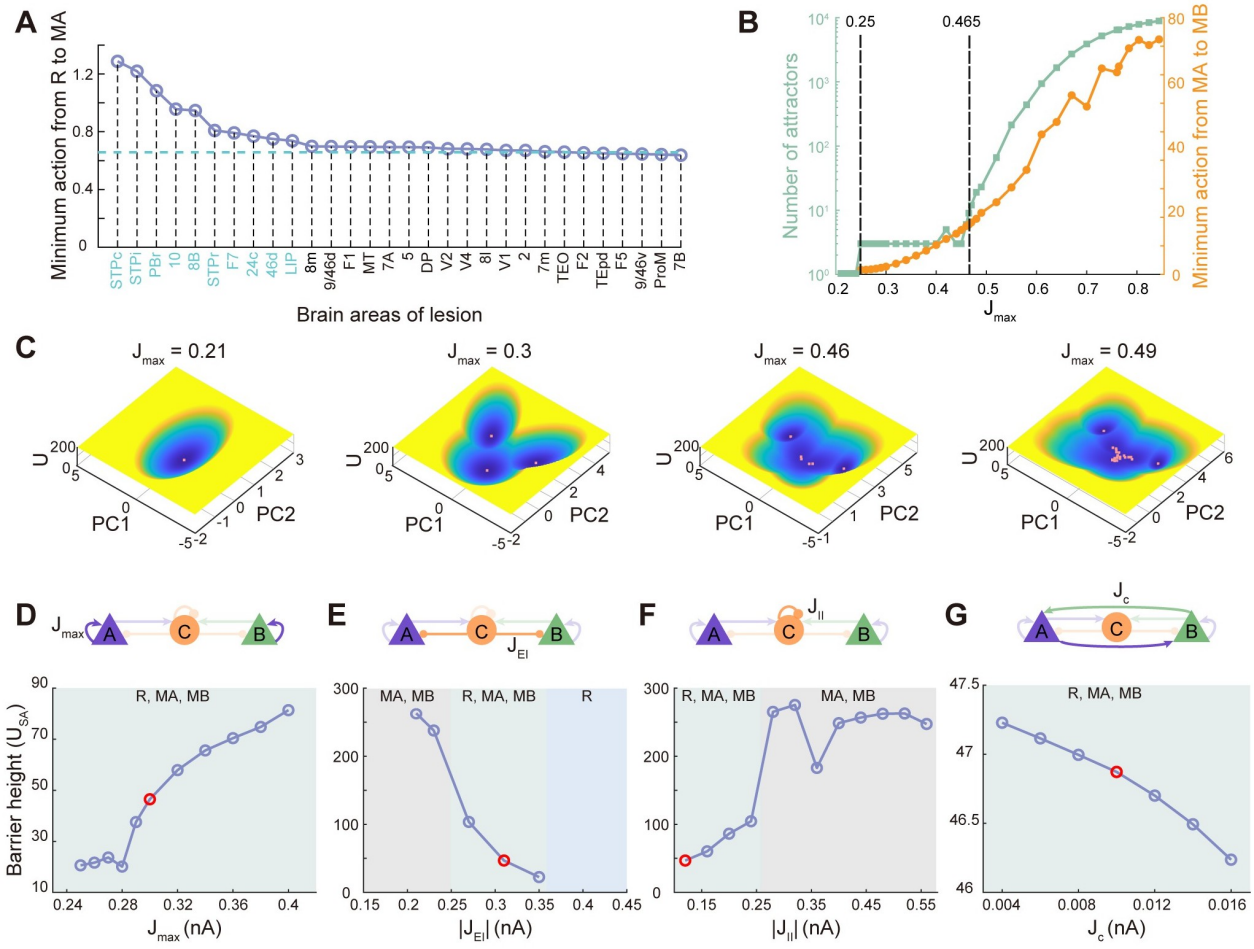
Connectivities in local circuits underlie working memory performance. (A) Effects of lesions on individual areas on transition action from spontaneous state to the memory state. The blue dashed line represents no brain lesions. The increase in the transition action for any silenced areas suggests that the formation of distributed working memory becomes harder. The top 10 silenced brain areas which have a strong impact on working memory are consistent with the ‘bowtie hub’ proposed by [33] except LIP. (B) Both the number of attractors and transition action from spontaneous state to memory state increase with increasing maximum recurrent strength (*J*_*max*_). (C) Four typical landscapes for increasing *J*_*max*_. (D-G) The robustness against random fluctuations as a function of the barrier height of MA (*U*_*SA*_), quantifying the network structure. The red dots represent default values.

To explore the roles of different brain areas on working memory function, we can quantify the influence of focal lesions on individual areas on the robustness of distributed working memory patterns by the minimum action from R to MA under no external stimulation. The silence of the brain area is conducted by cutting off its communication with other areas. The increase (decrease) of the transition action after silencing an individual area suggests that the formation of distributed working memory becomes harder (easier) after silencing the corresponding brain area, which demonstrates that this area plays a key role in maintaining the working memory state. The blue line in Fig 5A represents no brain lesions. We picked the top 10 key brain areas identified from the above procedure which display a strong impact on maintaining working memory function, and found that these 10 areas are consistent with the ‘bowtie hub’ proposed by [33]. Across all analyses performed above, we assumed the maximum area-specific recurrent synaptic strength *J*_*max*_ = 0.3, below the critical value needed for tristability in isolation (0.465). We explored the change of the number of attractors and minimum action from MA to MB as a function of *J*_*max*_ in Fig 5B (*J*_*min*_ is fixed as 0.21). The number of attractors is determined by initializing the DWM by 10000 initial conditions sampled from standard uniform distribution and counting the number of unique fixed points using a tolerance of 10%. There exists a lower limit of *J*_*max*_ (0.25 in our simulation) for the emergence of memory-related attractors (compare the first two panels in Fig 5C), highlighting the importance of the gradient of synaptic excitation in the neocortex. When *J*_*max*_ exceeds the threshold, larger *J*_*max*_ indicates that more brain areas high in the hierarchy have strong local reverberation to generate self-sustained persistent activity and be able to display multistability even isolated from the network. Therefore, it is reasonable that the large-scale circuit we studied here displays a large number of attractors for larger *J*_*max*_ (green line in Fig 5B). Some typical landscapes with multistable attractors are presented in Fig 5C (See Fig B in S1 Text for corresponding multiple stable states). These novel attractors potentially subserve internal processes and need to be validated by experiments in the future. Moreover, the minimum action from MA to MB tends to increase with respect to *J*_*max*_, demonstrating the more difficult state transition and thus stronger robustness of the memory state.

Human cognitive processes including working memory are influenced not only by external task demands but also by inherent fluctuations. During the delay period, if there is no external distractor, the robustness of working memory is influenced by the non-specific noise. The robustness against noise relies on both the magnitude of noise and the network structure. It is anticipated that a larger magnitude of noise leads to weaker robustness. In a previous study on the classic local circuit model of working memory, it is found that both increasing self-excitation and mutual inhibition are beneficial to the robustness of the working memory system to the random fluctuations [13]. However, for the distributed working memory model engaging multiple interacting brain areas we studied here, the role of network structure on robustness is more complex. In Fig 5D, 5E, 5F and 5G, we show the dependence of the barrier height of MA (*U*_*SA*_) on the network structure (the red dots represent the case for default modeling parameters), more specifically, on the maximum area-specific recurrent synaptic strength (*J*_*max*_), the inhibition strength from inhibitory population to excitatory populations (*J*_*EI*_), the self-inhibition strength of inhibitory population (*J*_*II*_) and the cross-coupling strength between excitatory populations (*J*_*C*_). The MA state with the higher barrier height (larger *U*_*SA*_) is more robust to the noise.

For *J*_*max*_, we focus on the situation where the landscape is tristable. We found that as *J*_*max*_ increases, the attractors representing memory states become deeper while the attractor representing resting state becomes shallower, leading to harder noise-driven switch from memory attractor to the resting one (Fig 5D). This is in line with the results from minimum action (orange line in Fig 5B) indicating larger transition action for larger *J*_*max*_. This is also consistent with the previous study based on local circuit [13]. The influence of *J*_*EI*_ is more complex (Fig 5E). For small *J*_*EI*_, the landscape exhibits two symmetric attractors encoding memory states. The additional increase of *J*_*EI*_ induces the emergence of the attractor representing the resting state. However, the two symmetric memory states disappear simultaneously for further increase of *J*_*EI*_, so the system loses its ability to perform working memory. The decaying *U*_*SA*_ as a function of *J*_*EI*_ suggests that weaker inhibition strength to excitatory populations is requisite for the emergence and strong robustness of memory states. Consistently, the self-inhibition of inhibitory populations is required to be stronger to reduce the inhibition to excitatory populations, thus enhancing the robustness of memory states (Fig 5F). Furthermore, smaller mutual excitation between two competitors (population A and B) is beneficial to the winner-take-all competition between the two competitors, explaining the declining *U*_*SA*_ for larger *J*_*C*_ (Fig 5G).

### Landscape control identifies the optimal combination of stimulation targets for improving the working memory function

**Fig 6:**
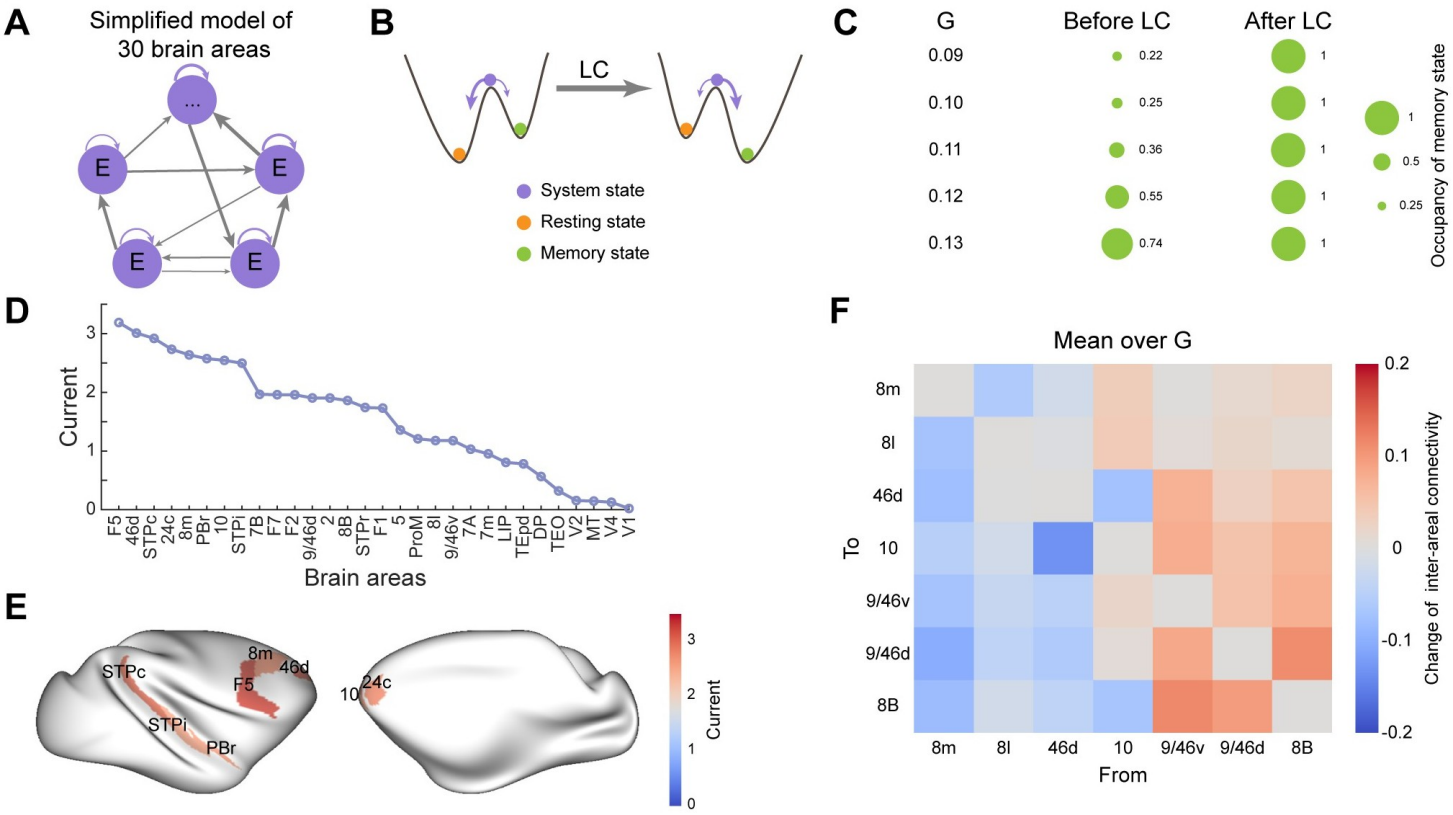
Landscape control identifies the optimal combination of stimulation targets for the improvement of behavioral performance. (A) Scheme of simplified distributed working memory model which is composed of 30 interconnected excitatory areas with a gradient of local coupling strengths. (B) An illustration of the landscapes of the simplified model before and after landscape control (LC). Before control, the resting state is deep and stable; after control, the memory state becomes dominant and the system is more inclined to stay in the memory state. (C) The change of the occupancy of memory state before and after LC under different global coupling strengths *G*. (D) The external stimulation currents of each brain area identified by LC for the improvements of working memory. (E) The top 8 optimal stimulation targets for the improvement of working memory function. The corresponding stimulation currents are indicated by colors. (F) By landscape control, the change of inter-areal connectivity in the prefrontal network can be identified to achieve larger occupancy of desired memory state.

The single-factor sensitivity analysis (Fig 5A) can be employed to explore the effects of individual brain area on the potential landscape. However, it is hard to identify the combined influence of multiple brain areas due to the large computational cost. Landscape control (LC) based on transition actions optimization provides an effective way to perform multi-factor analysis [44, 45]. In addition, previous evidence demonstrates that increased stability of brain state is accompanied by better working memory behavioral performance [57]. LC allows us to alter the stability of system states in desired ways by manipulating tunable parameters. Thus, we seek to identify the optimal combinations of stimulation locations of neuromodulation for the improvement of working memory function through increasing the stability of desired memory state. LC is implemented based on the simplified distributed working memory model (schemed in Fig 6A), which is comprised of 30 interconnected excitatory nodes with a gradient of local coupling strength (See Section B in S1 Text for details). The simplified model exhibits two stable states, identified as the resting state and memory state (Fig E in S1 Text). The goal of LC is to increase the stability of the memory state while decreasing the stability of the resting state, thus inducing the resting to memory state transition and achieving better behavioral performance (schemed in Fig 6B). After the transition action optimization (See Methods), the occupancy of memory state approaches 1 for different global coupling strength *G* (Fig 6C), suggesting the effectiveness of LC methods. Considering the heterogeneity of individuals in working memory capability, the optimal intervention is defined as the average over multiple realizations characterized by different *G*. The external currents applied into each brain area from optimization are shown in Fig 6D. The top 8 targets (Fig 6E) are consistent with the single-factor analysis (Fig 5A). Both strategies highlight the importance of associative areas, especially the prefrontal and parietal, in higher-order cognitive functions such as working memory. In contrast, in these strategies, the early sensory areas receive lower external currents. The optimal combination of stimulation targets identified by LC can be potential candidate locations of neuromodulation such as tACS and tDCS [58].

Learning involves finding synaptic weights to maximize rewards. Based on the large-scale distributed working memory incorporating dopamine, Froudist-Walsh *et al*. updated the strength of cortical-VTA connection via reinforcement learning and the correct timing of dopamine release is learned [59]. Landscape control can also be employed to predict the change of synaptic weights (FLN strength in our model) induced by learning to achieve better behavioral performance (larger reward). Since the prefrontal cortex is more crucial for working memory, we optimize the connectivity strength in the subnetwork comprised of 7 prefrontal areas (8m, 8l, 46d, 10, 9/46v, 9/46d, 8B) to increase the occupancy of desired memory state. After optimization, the connections originated from 9/46v, 9/46d, and 8B are strengthened while those from 8m, 8l, and 46d are weakened (Fig 6F, see Fig F in S1 Text for results under different global coupling *G*), suggesting more crucial contributions of the three areas (9/46v, 9/46d and 8B) to working memory function.

Our landscape control approach emphasized the role of specific regions within the dorsolateral prefrontal cortex (DLPFC), namely 46d, 9/46v, and 9/46d, in the functioning of working memory (WM). Extensive research has consistently implicated the DLPFC in WM processes [60, 61]. Due to its established involvement in WM, the DLPFC has been selected as the prime target for stimulation in various studies. These investigations have yielded noteworthy findings, demonstrating that stimulation of the DLPFC leads to significant improvements in reaction times among healthy individuals and enhanced response accuracy in both healthy and neuropsychiatric individuals [62].

The landscape control is a general approach and can be applied to other neural systems. For example, the induced transition from depressive state to healthy state through external stimulation may be a promising strategy for the therapy of major depressive disorder [63].

## Discussion

Working memory is associated with the stimulus-selective persistent activity after the withdrawal of stimulus. The storage of memory requires the robustness of persistent activity to inherent noise in brains and external distractors. The concept of attractor dynamics was proposed by previous theoretical works to describe the robustness of working memory [21, 23, 48]. In the attractor landscape or energy landscape framework, each possible stable state corresponds to a basin of attraction, and the stability of each stable state can be quantified by the depth of the basins. Different from traditional local approaches for studying the mechanism of working memory by tracking the neural activity trajectories in a limited time scale, the landscape approach provides a way to investigate the global stability of the working memory function. Recently, large-scale models engaging multiple brain regions have been proposed, which facilitate the investigation of the contributions of different brain regions to distributed functions [28]. However, how to visualize the landscape of the brain from a large-scale brain network remains a challenge.

In this work, based on an anatomically constrained distributed working memory model [28], we quantified the corresponding energy landscape with moment equation approximations [10, 38, 39] and visualized it with a dimension reduction approach [39]. The landscape provides a global picture of the stochastic dynamics of distributed working memory. In the absence of external stimulation, three attractors appear on the landscape, characterizing spontaneous state and two memory states encoded by two selective excitatory populations, respectively. The working memory function is governed by the change of landscape topography and switch of system state according to the task demands, which explains the neural activities trajectories from multiple brain regions. Therefore, the landscape framework fits well with the distributed working memory idea, since the attractor concept is indeed defined in a high-dimensional space involving the neural activities of multiple brain regions. Also, the noise-driven state transition from stimulus-related attractor to distractor-related attractor during the delay epoch accounts for the behavioral error.

In this investigation, two kinds of noises that may induce state transition are considered: non-selective random fluctuations and distractor stimuli. By defining the barrier height between stable states to quantify the landscape topography, we found that larger strength of distractor stimuli destabilizes stimulus-related attractors, leading to weaker robustness to distractors. For non-selective fluctuations, we explored the dependence of the robustness of the working memory state on the network structure, more specifically, the excitation and inhibition strengths in local circuits. We found that both stronger strength of self-excitation and self-inhibition are beneficial to the robustness of the working memory state while stronger strength of inhibition to excitatory populations and cross-coupling between excitatory populations have opposite effects. These results provide new insights into the design of network structure to achieve better behavioral performance of cognitive functions.

Moreover, the most probable biological path identified by the minimum action path approach reveals that the spontaneous state serves as an intermediate state during the switch between the two memory states, which may increase the plasticity of state transition. The memory stored in the cortical area with higher hierarchy is much more difficult to lose, and thus more stable. This agrees well with the hierarchy of intrinsic timescale across primate cortex [54, 55]. The increasing barrier height of stimulus-related attractors with time-evolving during the delay epoch also explains the mechanism of the temporal gating of distractors [29]. Interestingly, many novel attractors emerge during the increase of self-excitation, which may serve as various forms of internal representations. The functional role of these attractors warrants further explorations from both experimental and theoretical efforts.

To unravel the role of different brain regions in working memory, we performed single-factor sensitivity analysis. Specifically, we quantified the difficulty of transition from resting state to memory state by transition action after individual brain lesion. One of the biggest challenges in brain science is how to manipulate brain function by modulating different brain regions. This is challenging since the brain is inherently non-linear, which goes beyond the simplified linear network models used in classic network control theory. To address this issue, we developed a control approach (landscape control) using the non-linear large-scale model of the macaque brain. We aim to improve working memory function by modulating the switching behavior between stable states. We employed two strategies: simulating neuromodulation techniques through modifying external currents and simulating learning through modifying inter-areal connectivity. Our findings indicate that associative areas, especially prefrontal and parietal cortical areas, play crucial roles in high-order working memory function.

Although our findings are primarily based on the delayed match-to-sample task, we have also investigated different working memory tasks using landscape approach (Fig G in S1 Text). Specifically, in the delayed discrimination task, participants are required to determine which of the two stimuli, separated by a delay period, has a higher intensity. We explored this task by delivering simultaneous input to the population A and population B in the DWM model. We further define the relative barrier height (*U*_21_) based on landscape, which serves to quantify the relative stability between the two stimuli. Our results reveal that *U*_21_ tends towards zero when the intensities of the two stimuli are equal. Consequently, the sign of *U*_21_ becomes indicative of the comparison decision. A positive value of *U*_21_ suggests that *I*_1_ has a higher intensity, whereas a negative value of *U*_21_ implies that *I*_2_ possesses a higher intensity. It is noteworthy that our landscape and transition path approach can also be extended to other working memory tasks, although adaptations in the underlying mathematical models may be needed.

As a complex system, the brain exhibits distinct principles of organization operating at different scales and the large-scale activity of the brain is more than the trivial sum of its components [64]. Thus, biophysical models of large-scale neuronal activity and their dynamics are crucial for the understanding of cognitive function [65]. The energy landscape and transition path approach provides a new paradigm to study the dynamics of large-scale neural networks. Here, we explored the underlying transition dynamics of distributed working memory, which is general to many other cognitive functions such as decision-making [48, 66].

The theoretical insights gained from this work help us better understand the underlying mechanisms and dynamics of working memory. With a deeper understanding of the dynamics and critical nodes in working memory networks, researchers can develop interventions that specifically target and modulate these areas. Working memory deficits are often seen in individuals with neurological conditions, such as depression [67], schizophrenia [68] and Parkinson’s disease [69]. In the future, it is expected that the landscape approach can be applied to study the underlying mechanism of brain disorders. The insights gained from this research could be applied to develop rehabilitation strategies or neurorehabilitation protocols that specifically address working memory impairments in these populations. The landscape control approach could be helpful in developing therapeutic strategies for brain disorders by changing the relative stability of different brain states. By targeting the critical transitions in the landscape, clinical interventions could potentially facilitate recovery and improve functional outcomes.

The distributed working memory model we explored here is primarily built upon anatomical data from the macaque brain, encompassing FLN, SLN, and spine count. For human brain, multimodal neuroimaging techniques can be leveraged to obtain comprehensive data. These techniques include diffusion magnetic resonance imaging (dMRI) or diffusion tensor imaging (DTI) for assessing structural anatomical connectivity, functional magnetic resonance imaging (fMRI) for investigating functional connectivity, and positron electron tomography (PET) for examining neurotransmission (receptor density). By integrating these data sources, the whole-brain models of the human brain can be developed to explore the underlying mechanisms of brain dynamics and cognitive functions [52, 70, 71]. In light of this, it would be intriguing to apply the landscape and transition path approaches to the human whole-brain model, revealing novel information about the critical nodes or areas that exert significant influence over working memory stabilization.

## Supporting information

S1 Text Supporting information for “Controlling brain dynamics: Landscape and transition path for working memory”. Description of dimension reduction of multi-dimensional landscape and simplified computational model of distributed working memory, additional figures and tables.
(PDF)
