# Supplementary material for: Controlling brain dynamics: Landscape and transition path for working memory
Source: PLoS Comput Biol. 2023 Sep 5;19(9):e1011446. doi: 10.1371/journal.pcbi.1011446 (PMC10503743; doi:10.1371/journal.pcbi.1011446)
Supplement: S1 Text — Description of dimension reduction of multi-dimensional landscape and simplified computational model of distributed working memory, additional figures and tables. (PDF) [file pcbi.1011446.s001.pdf]

# Text S1: Supporting information for ”Controlling brain dynamics: landscape and transition path for working memory”

**Authors:** Leijun Ye[1,2], Jianfeng Feng[1,2,3*], Chunhe Li[1,2,4*]

**Affiliations: 1** Institute of Science and Technology for Brain-Inspired Intelligence, Fudan University, Shanghai, China

**2** Shanghai Center for Mathematical Sciences, Fudan University, Shanghai, China

**3** Department of Computer Science, University of Warwick, Coventry, UK

**4** School of Mathematical Sciences and MOE Frontiers Center for Brain Science, Fudan University, Shanghai, China

**E-Mail:** jianfeng64@gmail.com (J.F.), chunheli@fudan.edu.cn (C.L.)

## A. The dimension reduction of high-dimensional potential landscape

Inspired by the principle of Principal Component Analysis (PCA), we can reduce the dimensionality of the potential landscape but at the same time minimize information loss by projecting the system variables $\boldsymbol{X}$ into the new subspace spanning by the first few principal components [1]. More specifically, for the probability density function of the multistable system of Eq. 22 in the main text, the mean and variance of $\boldsymbol{X}$ are

$$\boldsymbol{\mu} = \sum_{j=1}^{M} w^j \boldsymbol{\mu}^j, \tag{1}$$

$$\boldsymbol{\Sigma} = \sum_{j=1}^{M} w^j (\boldsymbol{\Sigma}^j + \boldsymbol{\mu}^j (\boldsymbol{\mu}^j)^T) - \boldsymbol{\mu}\boldsymbol{\mu}^T. \tag{2}$$

Since the covariance matrix $\boldsymbol{\Sigma}$ is the positive semi-definite matrix, we can perform eigenvalue decomposition on $\boldsymbol{\Sigma}$. The eigenvectors corresponding to the first $C$ largest eigenvalues are denoted by $\boldsymbol{V} = (\boldsymbol{v}_1, ..., \boldsymbol{v}_C)$ with $\|\boldsymbol{v}_i\| = 1$ and $\boldsymbol{v}_i \boldsymbol{v}_j^T = 0$, $\forall i \neq j$. The projected system variable $\boldsymbol{Z}$ on the subspace spanning by $\boldsymbol{V}$ is denoted by $\boldsymbol{Z} = \boldsymbol{V}^T \boldsymbol{X}^j = (Z_1, ..., Z_C)$. The mean and covariance matrix of $j$th stable state after projection are $\boldsymbol{\mu}_z^j = \boldsymbol{V}^T \boldsymbol{\mu}_j$ and $\boldsymbol{\Sigma}_z^j = \boldsymbol{V}^T \boldsymbol{\Sigma}^j \boldsymbol{V}$, respectively. Correspondingly, the multi-dimensional normal distribution of $j$th stable state becomes

$$p_z^j(\boldsymbol{z}) = \frac{1}{(2\pi)^{\frac{C}{2}} \left|\boldsymbol{\Sigma}_z^j\right|^{\frac{1}{2}}} \exp\left\{ -\frac{1}{2} \left(\boldsymbol{z} - \boldsymbol{\mu}_z^j\right)^T \left(\boldsymbol{\Sigma}_z^j\right)^{-1} \left(\boldsymbol{z} - \boldsymbol{\mu}_z^j\right) \right\}. \tag{3}$$

The final probability density function after dimension reduction is $p_z(\mathbf{z}) = \sum_{j=1}^{M} w_j p_z^j(\mathbf{z})$, and the potential landscape is $U_z(\mathbf{z}) = -ln(P_z(\mathbf{z}))$. In this work, we choose $C = 2$, that is, the high-dimensional potential landscape is projected to the 2D subspace spanning by the first two components $PC1$ and $PC2$.

## B. Simplified computational model of distributed working memory

To identify the stimulation targets to improve working memory, we optimize the action to control the transition between resting state and memory state. However, the optimization based on the 90-D distributed working memory model is time-consuming and computationally expensive. Here, we describe a simplified model which preserves the hierarchy and connection structure of the original 90-D model and is suitable for the action optimization[2]. The simplified model is composed of 30 interconnected excitatory areas, each of them following a rate dynamics:

$$\tau \frac{dr_i}{dt} = -r_i + \phi(J\eta_i r_i + G\sum_{j=1}^{30} W_{ij} r_j + I_{bg} + I_{ext}).$$

Here, $r_i$ is the firing rate of area $i$. Each area has a relaxation time constant $\tau$ and receives a background input current $I_{bg}$. $J$ is the strength of self-connection and $G$ represents the global coupling strength. These parameters are chosen as $\tau = 20ms, I = 4.81$, and $J = 0.91$. $\eta_i$ is a monotonically increasing linear function: $\eta_i = \eta_{\min} + (\eta_{\max} - \eta_{\min})\, h_i$, introducing a gradient of connectivity strength across the network. $W$ is the connection weight matrix between areas estimated from FLN and takes the same values as the 90-D model. $\phi$ is the transfer function:

$$\phi(I) = \frac{S_{max}}{1 + e^{-S_{sat}(I - I_0)}},$$

where $S_{max} = 60$, $S_{sat} = 0.1$ and $I_0 = 30$. For these parameters, an isolated area is bistable only if $\eta \approx 0.88$. Thus, we choose $\eta_{\min} = 0.55$ and $\eta_{\max} = 0.85$ to guarantee that all areas are monostable in isolation. The currents $I_{ext}$ in each brain area are the manipulated parameters for landscape control. Furthermore, the visual cortex (V1) receives an additional current input (strength $I = 0.5$) to simulate the visual delayed response task.

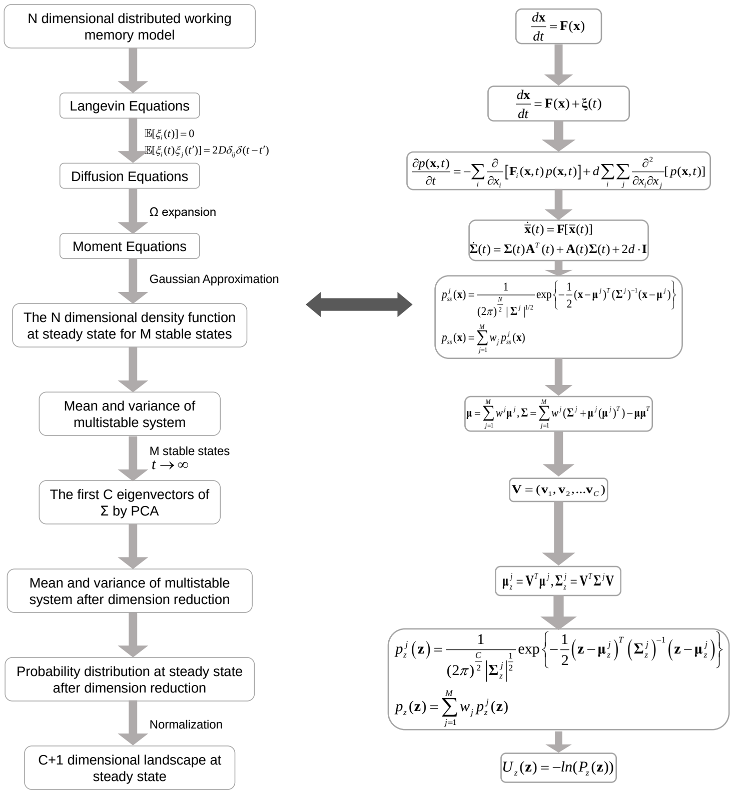


**Fig A.** Methodology flowchart for constructing the energy landscape from ordinary differential equations (ODEs) model of distributed working memory [1, 3, 4].

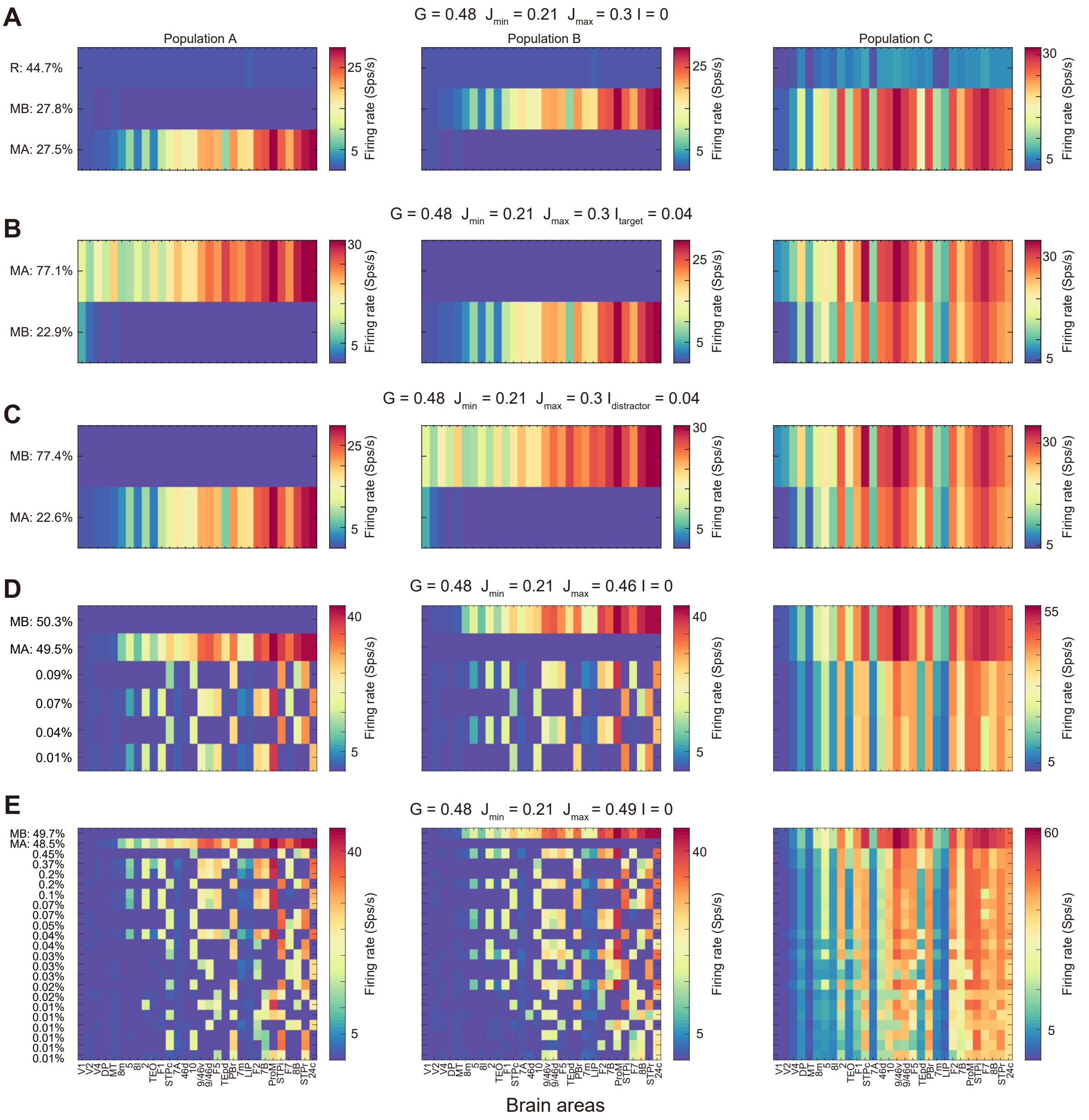


**Fig B.** Firing rate of each population at stable state under the different model setting. For each stable state, the following number indicates the statistic of frequency under 10,000 initial conditions. (A) Stable state when no stimulus is applied. (B) Stable states when a visual target stimulus (amplitude=0.04) is applied to population A in V1. (C) Stable states when a visual distractor stimulus (amplitude=0.04) is applied to population B in V1. (D-E) Stable states for larger maximum recurrent strength ($J_{max}$) and the system is multistable.

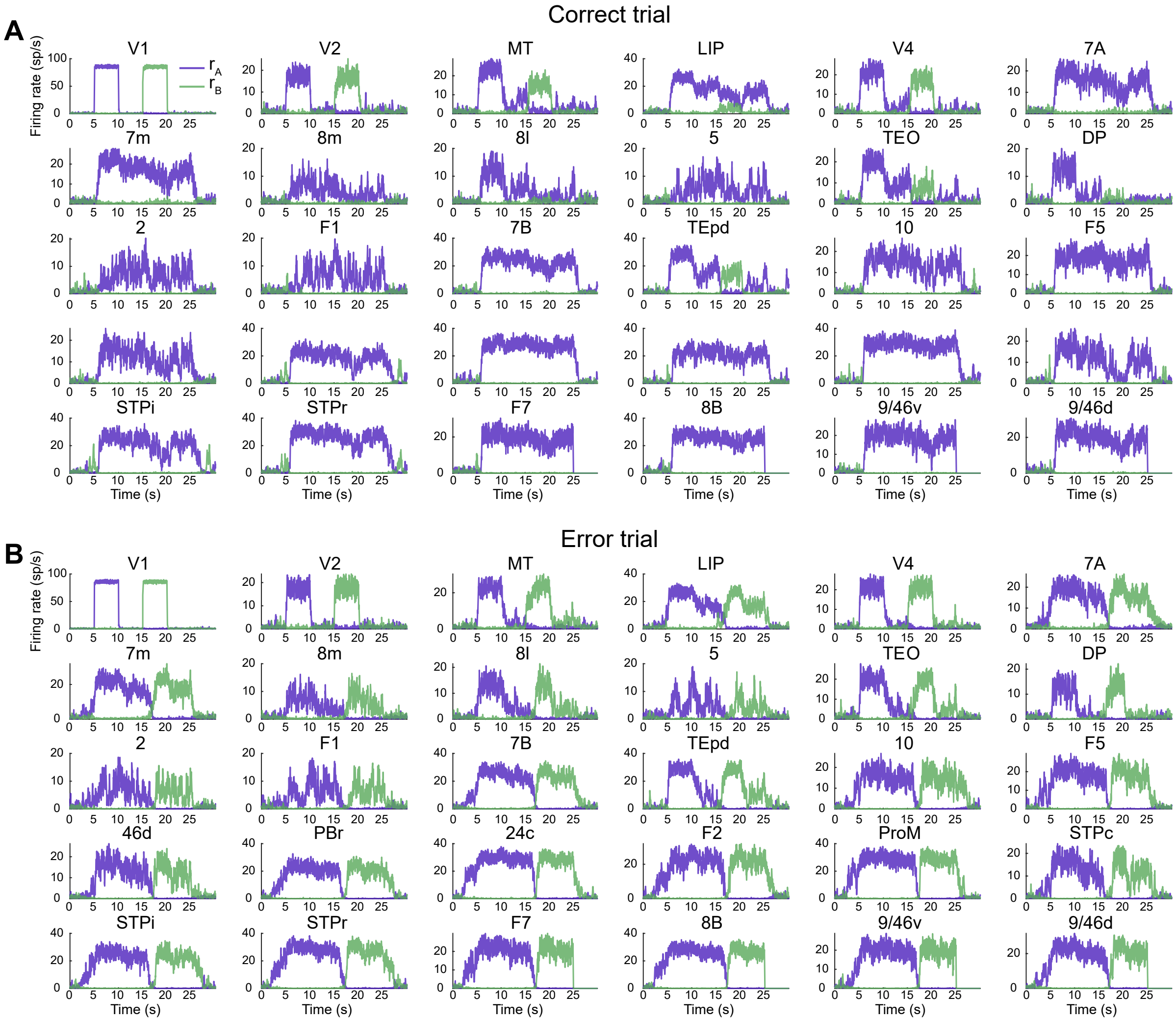


**Fig C.** (A) Time-evolving firing rate of populations A and B in 30 brain areas for the correct trial in the working memory task. (B) Same as (A) but for the error trial.

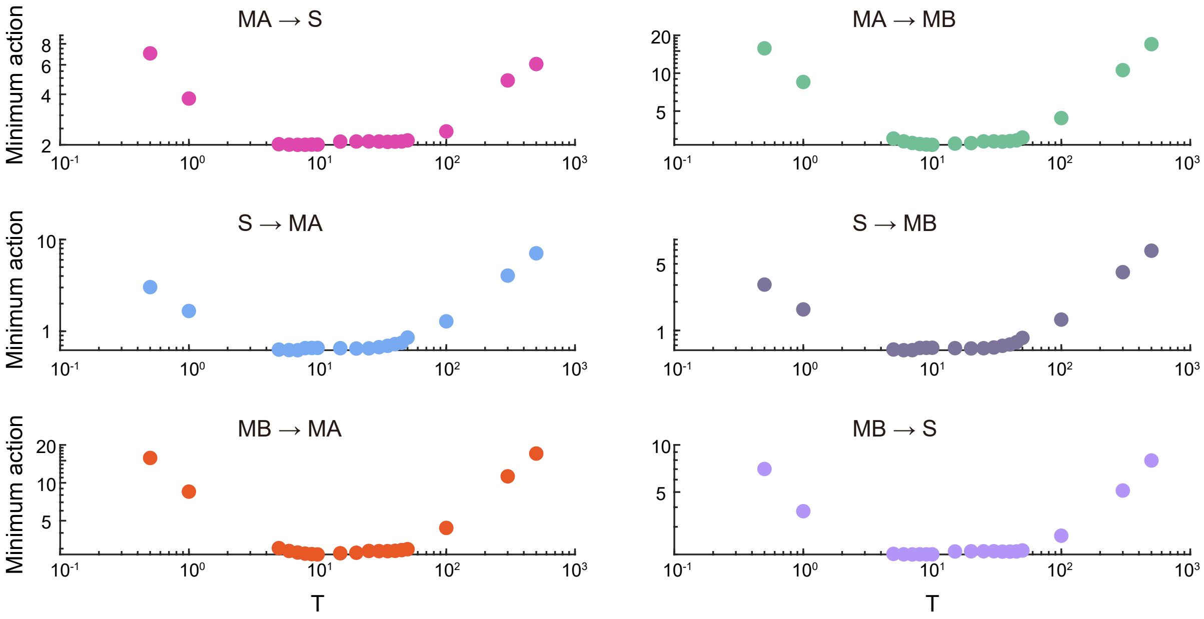


**Fig D.** The adaptive minimum action method is employed to obtain the minimum action path (MAP) numerically [5]. To find the optimal time interval $T$ for transition, we performed the grid search for different transition paths when the system is tristable with no external stimulus.

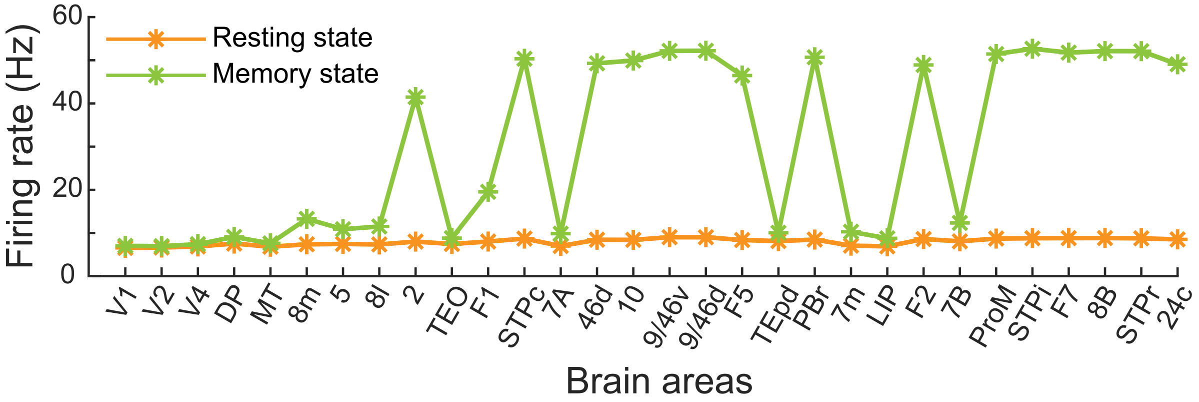


**Fig E.** Two stable states for the simplified model with $G = 0.12$.

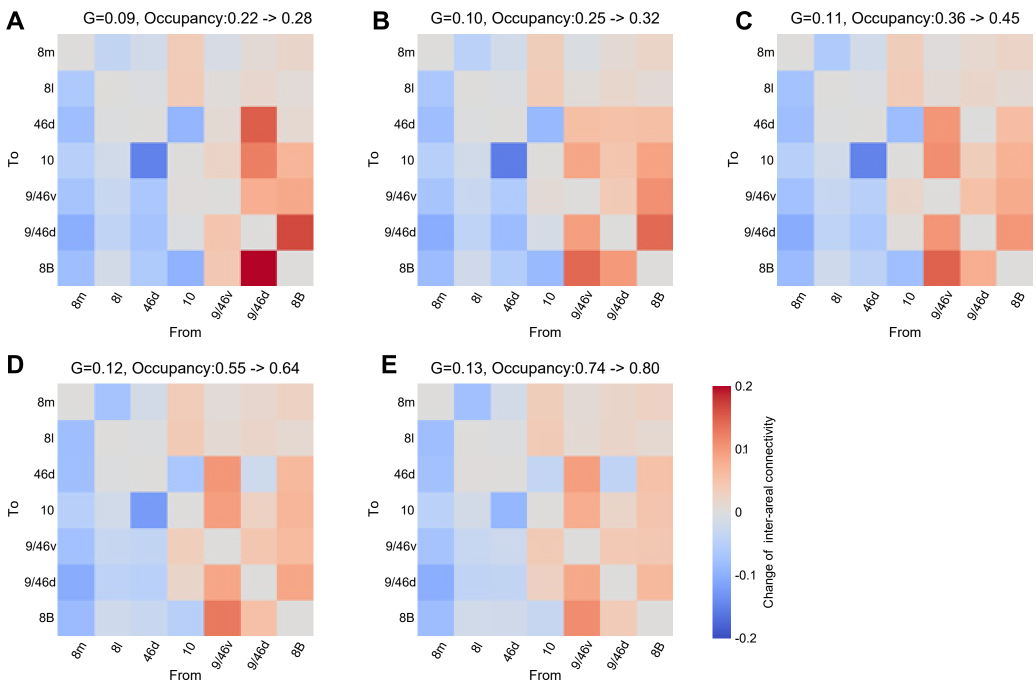


**Fig F.** The change of inter-areal connectivity between prefrontal areas after landscape control under different global coupling strengths *G*. The occupancy of memory state before and after landscape control is indicated on the top of each panel.

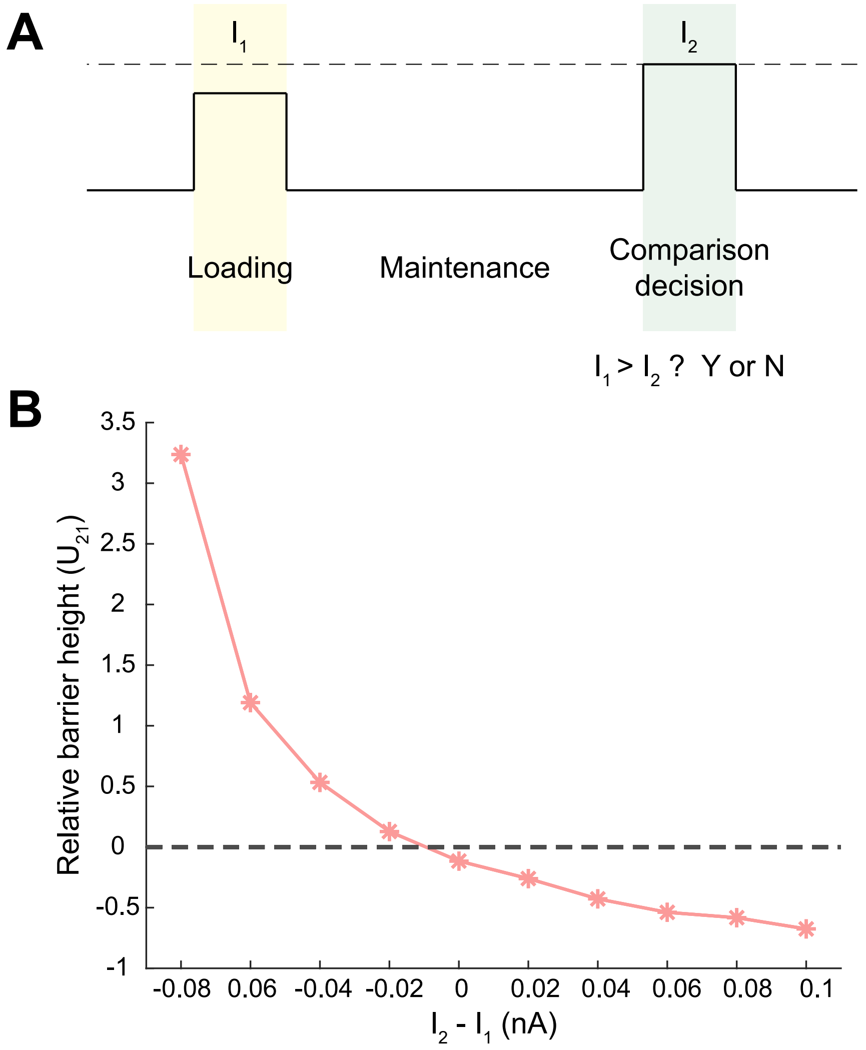


**Fig G.** (A) In the delayed discrimination task, participants are presented with a somatosensory stimulus of intensity $I_1$. After a delay period, another stimulus $I_2$ is shown, and the participants are required to determine whether $I_2$ is higher or lower than $I_1$. (B) Relative barrier height ($U_{21} = U_2 - U_1$) which refers to the potential difference between the local minima of the basin of attraction for $I_1$ and $I_2$ with respect to the difference in intensity between the two stimuli.

**Table A.** Spine count data from basal dendrites of layer 2/3 pyramidal neurons in young ( 2y o) macaque, acquired from the specified literature.

| Rank in SLN hierarchy | Area name | Measured spine count | Age correction factor | Source |
|---|---|---|---|---|
| 1 | V1 | 643 | 1 | [6, 7] |
| 2 | V2 | 1201 | 1 | [7] |
| 3 | V4 | 2429 | 1 | [8] |
| 4 | DP | - | - | - |
| 5 | MT | 2077 | 1 | [6] |
| 6 | 8m | 3200 | 1.30 | [8] |
| 7 | 5 | 4689 | 1 | [9] |
| 8 | 8l | 3200 | 1.30 | [8] |
| 9 | 2 | - | - | - |
| 10 | TEO | 4812 | 1 | [8] |
| 11 | F1 | - | - | - |
| 12 | STPc | 8337 | 1 | [6] |
| 13 | 7a | 2572 | 1 | [7, 10] |
| 14 | 46d | 6600 | 1.15 | Estimated from [11] |
| 15 | 10 | 6488 | 1.15 | [12] |
| 16 | 9/46v | 7800 | 1.15 | Estimated from [11] |
| 17 | 9/46d | 7800 | 1.15 | Estimated from [11] |
| 18 | F5 | - | - | - |
| 19 | TEpd | 7260 | 1 | [6] |
| 20 | PBr | - | - | - |
| 21 | 7m | 2294 | 1.30 | [13] |
| 22 | LIP | 2316 | 1 | [7, 10] |
| 23 | F2 | - | - | - |
| 24 | 7B | 6841 | 1 | [9] |
| 25 | ProM | - | - | - |
| 26 | STPi | 8337 | 1 | [6] |
| 27 | F7 | - | - | - |
| 28 | 8B | - | - | - |
| 29 | STPr | 8337 | 1 | [6] |
| 30 | 24c | 6825 | 1.15 | [14] |

**Table B.** Parameters used for modeling distributed working memory network.

| Parameter | Default Value | Description |
|---|---|---|
| $\tau_N$ | 60 ms | Time constant of NMDA receptor |
| $\tau_G$ | 5 ms | Time constant of GABAergic receptor |
| $\gamma_E$ | 1.282 | Kinetic parameter of excitatory population |
| $\gamma_I$ | 2 | Kinetic parameter of inhibitory population |
| $J_{min}$ | 0.21 | Self-excitation strength of excitatory populations for brain area with the lowest hierarchy |
| $J_{max}$ | 0.3 | Self-excitation strength of excitatory populations for brain area with the highest hierarchy |
| $h$ | − | Hierarchy of 30 brain areas |
| $J_S$ | $J_{min} + (J_{max} - J_{min})h$ | Self-excitation strength of excitatory populations in 30 brain areas |
| $J_C$ | 0.0107 nA | Cross-coupling strength between excitatory populations |
| $J_{EI}$ | −0.31 nA | Coupling strength from inhibitory population to excitatory ones |
| $J_{IE}$ | Area-specific | Coupling strength from the excitatory populations to the inhibitory one |
| $J_{II}$ | −0.12 nA | Self-inhibition strength of the inhibitory population |
| $I_{I0A}$ | 0.3294 nA | Background inputs to population A |
| $I_{I0B}$ | 0.3294 nA | Background inputs to population B |
| $I_{I0C}$ | 0.26 nA | Background inputs to population C |
| $a$, $b$, $d$ | 135 Hz/nA, 54 Hz, 0.308 s | Parameters for the transfer function of excitatory populations |
| $g_I$, $c_1$, $c_0$, $r_0$ | 4, 615 Hz/nA, 177 Hz, 55 Hz | Parameters for the transfer function of inhibitory populations |
| $G$ | 0.48 | Global coupling strength |
| $d$ | 0.1 | Diffusion coefficient |

# References


1. Kang X, Li C (2021) A dimension reduction approach for energy landscape: Identifying intermediate states in metabolism-emt network. Advanced Science 8: 2003133.

2. Mejias JF, Wang XJ (2022) Mechanisms of distributed working memory in a large-scale network of macaque neocortex. eLife 11: e72136.

3. Li C, Wang J (2014) Landscape and flux reveal a new global view and physical quantification of mammalian cell cycle. Proceedings of the National Academy of Sciences 111: 14130–14135.

4. Li C, Wang J (2013) Quantifying cell fate decisions for differentiation and reprogramming of a human stem cell network: landscape and biological paths. PLOS Computational Biology 9: e1003165.

5. Zhou X, Ren W, Weinan E, et al. (2008) Adaptive minimum action method for the study of rare events. The Journal of Chemical Physics 128: 104111.

6. Elston GN, Tweedale R, Rosa MGP (1999) Cortical integration in the visual system of the macaque monkey:

large-scale morphological differences in the pyramidal neurons in the occipital, parietal and temporal lobes. Proceedings of the Royal Society of London Series B: Biological Sciences 266: 1367–1374.

7. Elston GN, Rosa M (1997) The occipitoparietal pathway of the macaque monkey: comparison of pyramidal cell morphology in layer iii of functionally related cortical visual areas. Cerebral Cortex 7: 432–452.

8. Elston GN, Rosa M (1998) Morphological variation of layer iii pyramidal neurones in the occipitotemporal pathway of the macaque monkey visual cortex. Cerebral Cortex 8: 278–294.

9. Elston GN, Rockland KS (2002) The pyramidal cell of the sensorimotor cortex of the macaque monkey: phenotypic variation. Cerebral Cortex 12: 1071–1078.

10. Elston GN, Rosa MG (1998) Complex dendritic fields of pyramidal cells in the frontal eye field of the macaque monkey: comparison with parietal areas 7a and lip. NeuroReport 9: 127–131.

11. Elston G (2007) Specialization of the neocortical pyramidal cell during primate evolution. In: Kaas JH, editor, Evolution of Nervous Systems, Oxford: Academic Press. pp. 191-242.

12. Elston GN, Benavides-Piccione R, Elston A, Manger PR, Felipe Oroquieta Jd (2011) Pyramidal cells in prefrontal cortex: comparative observations reveal unparalleled specializations in neuronal structure among primate species. Frontiers Neuroanatomy : 2–2.

13. Elston G (2001) Interlaminar differences in the pyramidal cell phenotype in cortical areas 7m and stp (the superior temporal polysensory area) of the macaque monkey. Experimental Brain Research 138: 141–152.

14. Elston GN, Benavides-Piccione R, DeFelipe J (2005) A study of pyramidal cell structure in the cingulate cortex of the macaque monkey with comparative notes on inferotemporal and primary visual cortex. Cerebral Cortex 15: 64–73.
